# Sex-specific changes in energy demand during the preplaque stage in a transgenic Alzheimer’s mouse model

**DOI:** 10.1186/s13293-025-00737-0

**Published:** 2025-07-17

**Authors:** Rongwan Sun, Leonie-Kim Zimbalski, Stefanie Schreyer, David Baidoe-Ansah, Aida Harutyunyan, Arnd Heuser, Rachel N. Lippert, Joachim Spranger, Knut Mai, Sebastian Brachs

**Affiliations:** 1 Department of Endocrinology and Metabolism, European Reference Network on Rare Endocrine Diseases (ENDO-ERN), Charité – Universitätsmedizin Berlin, Corporate member of Freie Universität Berlin and Humboldt-Universität zu Berlin, Berlin, Germany; 2 German Centre for Cardiovascular Research (DZHK), Partner site Berlin, Berlin, Germany; 3 Department of Neurocircuit Development and Function, German Institute of Human Nutrition Potsdam-Rehbruecke (DIfE), Nuthetal, Germany; 4 NeuroCure Cluster of Excellence, Charité – Universitätsmedizin Berlin, corporate member of Freie Universität Berlin and Humboldt-Universität zu Berlin, Berlin, Germany; 5 Max Delbrück Center for Molecular Medicine in the Helmholtz Association (MDC), Berlin, Germany; 6 German Center for Diabetes Research (DZD e.V.), Neuherberg, Germany; 7 Department of Human Nutrition, German Institute of Human Nutrition Potsdam-Rehbruecke (DIfE), Nuthetal, Germany; 8 NutriAct-Competence Cluster Nutrition Research Berlin-Potsdam, Nuthetal, Germany; 9 Department of Endocrinology and Metabolism, Max Rubner Center for Cardiovascular Metabolic Renal Research (MRC), Charité – Universitätsmedizin Berlin, Hessische Straße 3-4, 10115 Berlin, Germany

**Keywords:** Sex difference, Energy metabolism, Hepatocytes, Adipocytes, Mitochondrial respiration, Alzheimer’s disease, Preplaque stage, Mortality risk

## Abstract

**Background:**

Cognitive deficits and brain glucose hypometabolism, lipid peroxidation and mitochondrial dysfunction are early pathological events in murine models and patients with Alzheimer’s disease (AD). Data from our previous research indicate that transgenic mice of the APP23 line, a murine AD model, exhibited higher energy expenditure and mitochondrial dysregulation in the liver as early as 3 months of age, which is considered the preplaque stage. Since women have a higher risk and mortality rate for AD, with potential sex-specific confounders as longevity, biological, genetic, and social factors also needing to be considered, sex differences in energy metabolism in AD remain insufficiently investigated.

**Methods:**

Here, we investigated sex-specific differences in mitochondrial respiration and metabolic profiles of 3–4-month-old, preplaque APP23 transgenic mice, in which we did not detect inflammatory signals and pathological amyloid-beta (Aß) plaques in brain or liver. Their mitochondrial respiration was assessed measuring oxygen consumption rates in isolated primary hepatocytes, stromal vascular cells (SVCs) and re-differentiated adipocytes. Furthermore, we analyzed energy balance, including food intake, locomotor activity, energy expenditure and fecal calorie loss.

**Results:**

We observed an upregulation of hepatic mitochondrial respiration in preplaque APP23 females. Female-derived SVCs and differentiated adipocytes improved mitochondrial flexibility with palmitate loading in vitro, which was in line with decreased plasma triglycerides in preplaque APP23 females in vivo. However, no differences in mitochondrial respiration were detected in hepatocytes and re-differentiated adipocytes derived from male APP23 mice. Furthermore, we corroborated an increased mortality during the preplaque stage, particularly in females, which exhibited reduced hyperactivity and caloric intake before death compared to survivors.

**Conclusions:**

Our data demonstrate that preplaque APP23 female mice have disequilibrated mitochondrial oxidation in hepatocytes and adipocytes as well as higher energy expenditure due to increased activity before AD manifestation. In contrast, male APP23 mice did not exhibit such metabolic changes. Constant excessive energy loss and limited calorie supply potentially contribute to the higher risk of mortality, especially in APP23 females during young adulthood.

**Plain english summary:**

Alzheimer’s disease (AD) affects men and women differently, with women at higher risk and mortality. This study explored sex differences in energy metabolism using APP23 transgenic mice, a model of AD, at young age (3–4 months) - before pathological amyloid-beta (Aß) plaques develop in the brain and liver. Female APP23 mice showed increased mitochondrial activity in liver and fat cells, higher energy expenditure, and more movement while eating less. They also excreted more energy in their feces. Notably, female APP23 mice had a lower survival rate than males. Before death, they became less active and ate even less, suggesting an inability to maintain energy balance. These findings indicate that female APP23 mice experience excessive energy loss, which may contribute to early mortality. Understanding these sex-specific metabolic differences could provide new insights into AD progression and highlight the need for targeted treatments.

**Supplementary Information:**

The online version contains supplementary material available at 10.1186/s13293-025-00737-0.

## Highlights

AD-related inflammatory signals and pathological Aβ plaques are virtually absent in the brain and liver of young APP23 mice in the preplaque stage.

Preplaque APP23 transgenic female mice upregulate hepatic mitochondrial respiration, which is not observed in APP23 males.

Ex vivo differentiated adipocytes isolated from preplaque APP23 females exhibit increased mitochondrial flexibility to fatty acid-induced metabolic stress.

The preplaque APP23 females show increased activity with lower diet intake, while both sexes of APP23 mice have higher fecal energy loss compared to wild-type controls.

The negative energy balance could contribute to the increased premature mortality of young APP23 mice in the preplaque stage.

**Supplementary Information:**

The online version contains supplementary material available at 10.1186/s13293-025-00737-0.

## Graphical Abstract

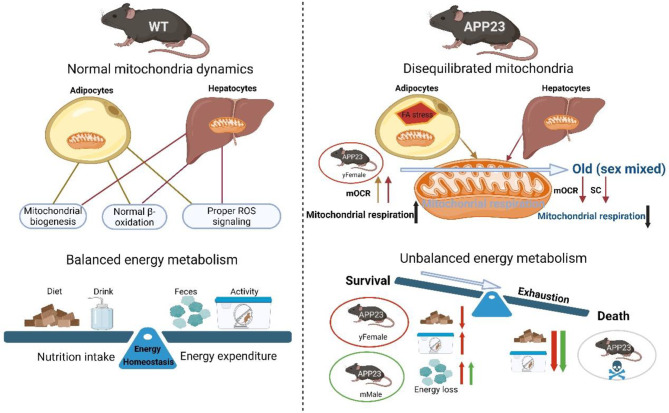

## Background

Alzheimer’s disease (AD) is characterized by neurodegeneration leading to progressive cognitive decline. Approximately 60–70% of dementia cases are attributed to AD [1]. The mortality of AD increased rapidly in the United States, with experts predicting 42.2 deaths per 100,000 people within the year 2023 [2]. The burden of AD-related deaths implies the need for early diagnosis and interventional therapies.

Approximately two-thirds of AD patients are female [3]. Sex differences in AD have been observed in clinical, neuroimaging, and pathology studies [4]. Women’s higher AD prevalence is linked to survivor bias in men [5], stronger biological risk factors like the APOE4 allele, and more prevalent psychiatric conditions such as depression [6]. Pregnancy-related complications and lower cognitive reserve also contribute to their increased risk [7]. Elderly patients with AD frequently have various comorbidities, such as hypercholesterolemia, hypertension, cardiovascular disease, atherosclerosis, depression and diabetes, while diabetes has been identified as a significant and common risk factor for AD progression [8], presumably through the accumulation of advanced glycation end products in neurofibrillary tangles and amyloid-β (Aβ) plaques in the brain [9]. In turn, 80% of AD patients develop glucose intolerance, insulin resistance or type-2 diabetes [10]. Moreover, diabetics often show reduced cognitive performance [11].

Although Aβ plaques and tau protein hyperphosphorylation are neuropathological hallmarks of AD, the toxicity of Aβ oligomers potentially inducing pro-inflammatory responses in microglia has already been demonstrated in earlier stages of AD [12]. The latter is hypothesized to be a reason for early symptoms in AD. Evidence indicates that mitochondrial dysfunction arises in mouse models and patients with obesity, insulin resistance, diabetes [13] and especially AD [14–18]. Therefore, further research on the contribution of mitochondrial function is vital to better understand the pathomechanisms involved in the earlier stages of AD.

Mitochondria play an essential role in all cells, especially in neurons, as the brain consumes 20% of the body’s glucose while only accounting for 2% of the body’s weight [19]. Cells use metabolites and enzymes to maintain the tricarboxylic acid cycle and oxidative phosphorylation (OXPHOS) to ensure ATP production. In addition, mitochondria perform multiple functions, including reactive oxygen species (ROS) production, cooperation in the endoplasmic reticulum (ER) stress response or unfolded protein response (UPR) and Ca^2+^ flux regulation. Mitochondrial dysfunction may affect glucose and lipid metabolism, resulting in energy imbalance.

We previously reported that early changes occur in liver and adipose tissue prior to Aβ plaque deposition in the brain, influencing whole-body metabolism in the APP23 transgenic AD mouse model (APP23) [20], which overexpresses the human *APP751* gene containing the Swedish double mutation [21]. In APP23, the first onset of cognitive deficits begins at 3 months of age [22]. These impairments included decreased performance in water maze setups [23–26] and reduced passive avoidance learning [27, 28], but without recognizable plaque formation. During the preplaque stage, increased mortality rates were observed in APP23 mice, which were sex-specifically elevated in females [29]. However, dense-core Aβ plaques [21, 27, 30], surrounded by microglia for clearance [31], hyperphosphorylated tau, dystrophic neurites and axonal sprouting [21, 27], and inflammatory responses [21, 27, 32, 33] were only observed in mice from 6 months of age. The liver represents an important organ for Aβ pathology in AD as a major source of circulating Aβ crossing the blood-brain barrier [34]. Moreover, hepatic enzymes catabolizing Aβ plaques are reduced in AD patients [35, 36].

Our previous proteome data revealed changes in mitochondrial proteins in the liver of preplaque APP23 females [20]. Based on this, we chose 3–4-month-old, male and female APP23 mice to study hepatic mitochondrial respiration. Furthermore, lipid metabolism and possible sex-specific differences in the general metabolic phenotype were addressed. Finally, we suggest a new perspective on the occurrence of increased mortality in preplaque male and female APP23 mice.

## Methods

### Animals

All experiments were performed in accordance with Guidance on the operation of the Animals (Scientific Procedures) Act 1986 and associated guidelines, EU Directive 2010/63 and complied with institutional ethical and ARRIVE guidelines and were approved by Landesamt für Gesundheit und Soziales Berlin (T0180/16) and Forschungseinrichtung für Experimentelle Medizin Charité (T-CH19/21). Additionally, previously collected data and stored samples from a prior experiment (G0074/16) were analyzed. Male and female mice were used for all experiments as this study particularly focused on sex-specific differences and therefore sex was investigated as a critical biological factor. Analyses of sex differences are summarized in Supplementary Tables 1 and/or indicated within the results.

**Fig. 1:**
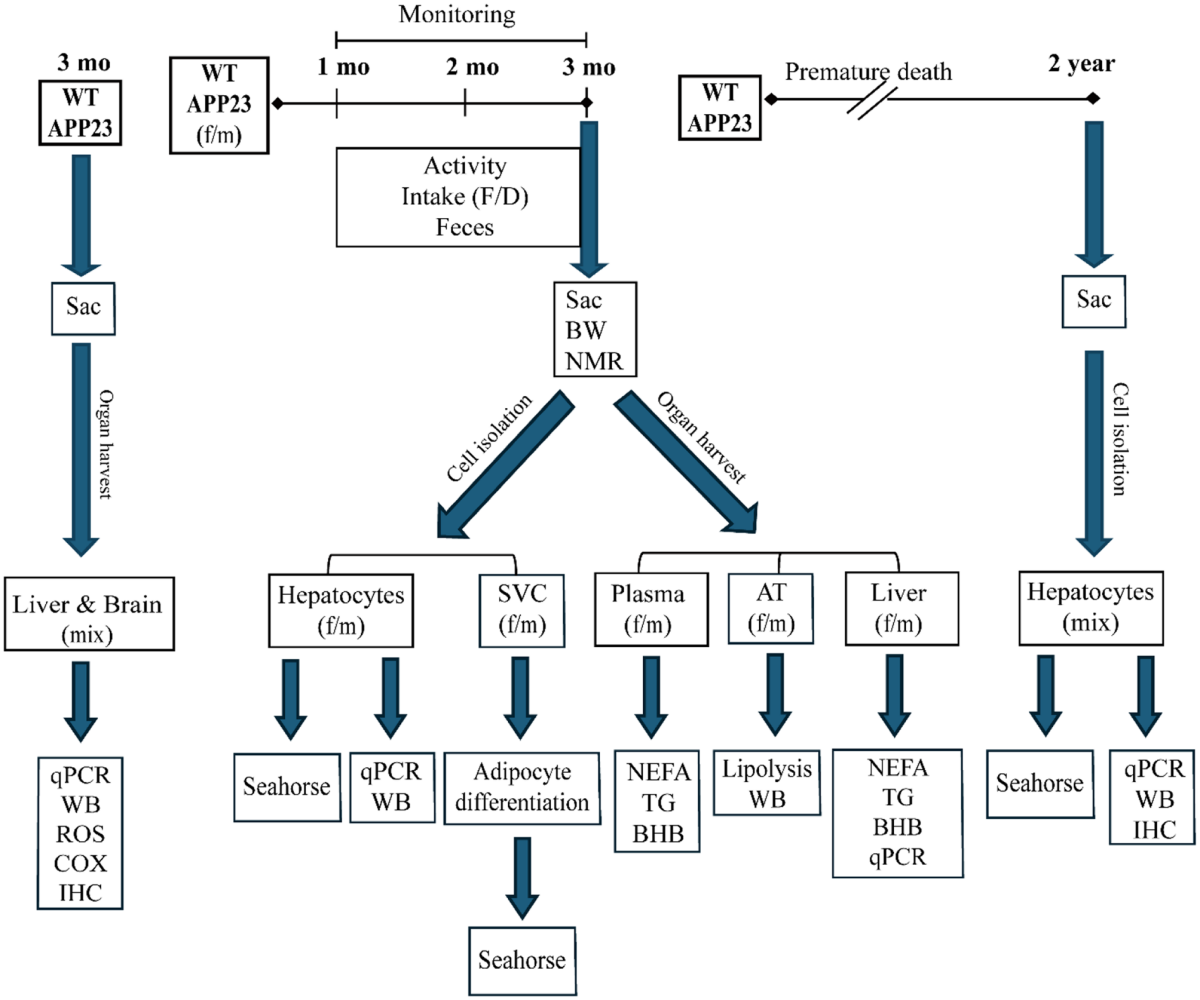
Schematic overview of the experiment design. APP23: APP23 transgenic mice, AT: adipose tissue, BW: body weight, BHB: beta-hydroxybutyrate. COX: total COX enzyme activity, D: drink, ROS: Reactive oxygen species evaluation by 2′,7′-dichlorofluorescein diacetate-detection, f: female, F: food, IHC: immunohistochemistry, mo: month, m: male, mix: sex-mixed, NEFA: Non-esterified fatty acid, qPCR: quantitative RT‒PCR, Seahorse: OCR and ECAR measurements, Sac: sacrifice, SVC: stromal vascular precursor cell, TG: triglyceride, WT: wild-type mice, WB: western blots

As described [20], the APP23 line was maintained on a C57BL/6J background by breeding transgenic APP23 males with wild-type (WT) females. Pups were weaned and genotyped at 3–4 weeks of age and littermates were used in all experiments. Mice had ad libitum access to normal diet (ND, Supplementary Table 2) and water, and both were measured weekly. Mice were predominantly pair-housed with a 12-hour light/dark cycle in individually ventilated cages or in the Digital Ventilated Cage system (DVC, Tecniplast), which continuously monitors intracage activity. Activity data were analyzed by averaging the cage activity index (from DVC) over the number of mice in the cage, which were housed separately to group only same genotypes. The data of surviving APP23 males and females were compared to those of dying mice (premature deaths) of the same age range by retrospective analysis of the recorded data over 10 days prior to death. 3–4-month-old (young) mice were sacrificed, body composition was determined by ^1^H-magnetic resonance spectroscopy (NMR) using a Minispec LF50 Body Composition Analyzer (Bruker) and thereafter, plasma and organs were collected for further analyses. The design for this study is outlined as flowchart in Fig. 1 and detailed information about experiment assignment, genotype, sex and sample size are summarized in Supplementary Table 3.

### Isolation of primary hepatocytes

Hepatocytes were isolated from liver using the collagenase perfusion method. Young and 1.5-year-old mice were sacrificed and the abdominal cavity was exposed. Liver perfusion involved vena cava cannulation, opening of vena portae, and sequential perfusion with EDTA and type IV collagenase in EBSS solution. The liver was processed in 4.5 g/L D-glucose DMEM, filtered and purified with a Percoll gradient. After confirming ≥ 80% purity, 9000 hepatocytes/well were seeded into XF96 microplates, cultured, and starved the following day with 1 g/L D-glucose DMEM for 24 h. Measurements were conducted on the third day post-isolation, as detailed in Sect. 2.4. Cell culture media, materials and kits are listed in Supplementary Table 4.

### Isolation of primary adipocytes and stromal vascular cells (SVCs) for in vitro differentiation

Primary adipocytes were isolated from 250 mg of epigonadal white adipose tissue (eWAT) as described [37]. Minced eWAT was digested with fresh collagenase type II (Sigma) in pre-warmed Krebs-Ringer-Phosphate-HEPES (KRPH: 20 mM HEPES, 5 mM KH2PO4, 1 mM MgSO4, 1 mM CaCl2, 136 mM NaCl, 4.7 mM KCl; pH 7.4) for 30 min in a shaking water bath at 37 °C with 120 rpm and pipetting every 7 min for disruption. Digested cells were filtered through a 250 μm cell strainer (Thermo) followed by separation of adipocytes and SVCs via centrifugation (300g, 5 min, RT). Adipocyte top layer was transferred for further use (see Sect. 2.5), and pelleted SVCs were resuspended and cultured in DMEM supplemented with 4.5 g/L D-glucose. A total of 8000 SVCs/well were seeded into a XF96 cell culture microplate. After reaching 100% confluence, SVCs were differentiated into adipocytes with 500 µM IBMX, 1 µM dexamethasone, and 10 µg/ml insulin in medium for 2 days, with 10 µg/ml insulin in medium for another 2 days and cultured in medium for lipid accumulation until day 10. Differentiated adipocytes were treated with 167 µM palmitate-BSA (PA, 6:1 in 150 mM NaCl) or 28 µM BSA (control, in 150 mM NaCl) 1 h before measurements.

### Measurements of oxygen consumption rate (OCR) and extracellular acidification rate (ECAR)

Hepatocytes and adipocytes were incubated in assay medium in a CO₂-free incubator for 1 h calibration. Mitochondrial respiration was assessed using a Seahorse XFe96 Analyzer (Agilent) in real-time at 37 °C according to the Cell Mito Stress Test protocol. Briefly, OCR and ECAR were measured in 4 steps with 3 cycles each, including basal and inhibitor injections of 3.0 µM oligomycin, followed by 0.5 µM FCCP and finally 0.5 µM antimycin A and 1.5 µM rotenone (all Sigma). Standardized protocols were developed based on titration experiments. Spare capacity (SC) was calculated as the difference between maximal and basal OCR, indicating the organ’s adaptive capacity under metabolic stress. ATP turnover rate was calculated by subtracting proton leak from basal OCR. ECAR measurements reflected medium acidification due to proton release during glucose metabolism. Afterwards, Hoechst 33342 stain (Thermo) was used to normalize measurements to (living) cell quantity.

### Lipolysis, triglyceride, beta-hydroxybutyrate and liver parameter measurements

Freshly isolated adipocytes (see 2.3.) were deprived of substrates in glucose-free, 2% fatty acid-free BSA KRPH for 30 min and stimulated with 0, 10, 50, 100, or 1000 nM isoprenaline (Sigma) for 3.5 h. Non-esterified fatty acid (NEFA) release from the buffer under the adipocyte layer was measured by an NEFA-HR(2) kit (Wako) in duplicate. Triglycerides (TG) were measured from plasma or liver samples as described [20] using the Triglycerides FS 10’ kit (DiaSys). Beta-hydroxybutyrate (BHB) was measured in plasma and protein lysates from liver samples using the β-Hydroxybutyrate (Ketone Body) Colorimetric Assay Kit (Cayman Chemical) according to manufacturer’s protocol and normalized to used liver tissue (g). Liver parameter were measured in plasma on an AU480 chemistry analyzer (Beckman Coulter) according to instructions.

### Total COX activity and DCFDA ROS detection

Freshly prepared, immediately snap-frozen liver and sagittal 1/3 of right brain hemisphere samples from young mice were lysed in 1% Nonidet P-40/PBS using a cooled SpeedMill Plus homogenizer (Analytik Jena), centrifuged and aliquoted for short-term storage at -80 °C.

To assess total COX activity, 340 µg liver and 150 µg brain lysates were used in the Cyclooxygenase (COX) Activity Assay Kit (Abcam) according to the manufacturer’s instructions determining enzyme activity of all COX variants. COX activity was normalized to total protein concentration of the samples determined by BCA assay (Thermo). For ROS detection, a new aliquot was thawed on ice and 100 µg liver and 50 µg brain lysates were incubated with 2´,7`-dichlorofluorescein diacetate (DCFDA, Sigma) to measure reactive oxygen and nitrogen oxide species (ROS and RNOS) levels. Oxidized 2’,7’-dichlorofluorescein was detected on an Infinite M200 Pro (Tecan) at 485/520 nm [38]. Results were normalized to total protein concentration and hydrogen peroxide was used as standard.

### Bomb calorimetry

Feces were collected from bedding of females (age: 27 ± 1 weeks) that were single-housed for 48 h for indirect calorimetry at week 20 of different dietary interventions [20] and from mostly single-housed males (age: 20 ± 1 weeks) for 48 h. Fecal samples were weighed to assess total amount of feces and stored at -80 °C until bomb calorimetry. Samples were dried for 48 h at 60 °C, weighed, and approximately 250 mg was pelleted with 800 mg of benzoic acid powder (Roth) as burning aid. They were measured on a 6200 Isoperobol Calorimeter with a 6510 Water Handling System 6510 (Parr Instrument Company) according to manufacturer’s instructions. Fecal energy density was analyzed in cal/g feces, and the produced fecal mass was calculated in g/d.

### Quantitative RT‒PCR

RNA was isolated from snap-frozen tissue samples using TRIzol with DNase digestion. 1 μg of RNA was transcribed into cDNA by Revert Aid Reverse Transcriptase (Thermo). Quantitative RT‒PCR was performed on a LightCycler 96 (Roche) with GoTaq qPCR Master Mix (Promega). Relative fold change in gene expression was calculated by the 2^−ΔΔCT^ method with Peptidylprolyl isomerase A (*Ppia*), ribosomal protein L19 (*Rpl19*) and 18 S ribosomal RNA (*18 S)* as housekeeping genes. The sequences of primers used are listed in Supplementary Table 5.

### Western blot

Isolated primary hepatocytes or frozen tissue samples were homogenized in 250 mM saccharose, 20 mM HEPES, and 1 mM EDTA buffer using the SpeedMill. According to protein concentration assessed by BCA assay and depending on tissue, 10–30 µg of protein lysates were separated by 10% SDS‒PAGE and transferred onto a nitrocellulose membrane. Membranes were blocked with 5% skim milk/TBST for 1 h and incubated with Total OXPHOS Rodent WB Antibody Cocktail (Ab110413, Abcam), anti-GAPDH (2118, Cell Signaling), anti-PGC1a (2178, Cell Signaling), anti-NF-κB p50 (sc-8414, Santa Cruz) and anti-NF-κB p65 (sc-8008, Santa Cruz) o/n at 4℃. Membranes were probed with HRP-conjugated anti-mouse IgG (7076, Cell Signaling) or anti-rabbit IgG (7074, Cell Signaling), developed with enhanced chemiluminescence (ECL) substrate (Bio-Rad), and imaged on a ChemiDoc XRS+ (Bio-Rad). For western blots for oxidative stress (oxiblots), liver and brain lysates from young male and female mice were probed with an anti-DNP (dinitrophenylhydrazone) antibody (D9656, Sigma-Aldrich) to detect non-enzymatic carboxylation of proteins as a result of RNOS as described [39]. Optical density of blots was quantified using Image Lab (Bio-Rad) normalized to adequate loading controls.

### Histology

The identical liver lobe and left hemisphere of brain of 3–4-month-old mice was immediately fixed in 4% PFA at 4 °C for 24 h, dehydrated in 10% and 30% sucrose for 24 h each and frozen in 2-methylbutane on dry ice. Samples were embedded in O.C.T. Compound (Sakura) and 30 μm sections were cryocut using a CM1950 (Leica). Representative sections of CA1 segment of hippocampus and left liver lobe were washed in KBPS (3 × 10 min), blocked against autofluorescence with 0.3% glycine/KPBS for 5 min, permeabilized with 0.03% SDS/KPBS for 10 min and blocked again in 3% donkey serum/0.125% Triton X-100/KPBS for 1 h. Primary rat anti-CD68 (MCA1957, Bio-Rad) were incubated in signal stain solution (Cell signaling) at 4 °C o/n. After washing (3x, 10 min), secondary anti-rat IgG-CF633 (SAB4600133, Merck) were incubated for 1 h at RT, washed (3x, 5 min) in KPBS and 40% EtOH/PBS for 30 s. Aβ plaques and tau fibrils were stained with 1 µM methoxy-X04 (Merck) in 40% EtOH/PBS for 30 s, washed in 40% and 90% EtOH/PBS for 1 min each, dried and mounted (Immu-Mount, Thermo). Images were taken at 10x and 20x magnification with 305 nm and 594 nm on an Axio Observer 7 (Zeiss) and processed in ImageJ (V1.48).

### Statistics

Statistical analyses were performed and graphs were generated in Prism 9 (GraphPad). All data are presented as the mean ± SEM for curves or box plots (25th to 75th percentiles) with median and whiskers from minimum to maximum. The normality of the distribution of the data was analyzed by the Shapiro‒Wilk test, and the equality of variances was analyzed by Levene’s test. According to data distribution, unpaired two-tailed t test with Welch’s correction was performed to compare the differences between two groups, and independent multiple t test without correction for multiple comparisons was used if applied. Two-way ANOVA adjusted with Bonferroni multiple comparisons test for post hoc analysis or Welch and Brown-Forsythe one-way ANOVA with Dunnett’s T3 multiple comparisons test were used for more groups or repeated measures, respectively, depending on normal distribution. Sample size (*d*) is given for each experimental group (Supplementary Table 3). Differences between the sexes of the same genotype were analyzed using a three-way ANOVA with Bonferroni multiple comparisons test (Supplementary Table 1). For key experiments, effect size is included to support statistical analyses. Statistical significance was set as *p ≤ 0.05, **p ≤ 0.01, ***p ≤ 0.001.

## Results

### Old APP23 mice exhibit mitochondrial dysfunction and upregulated ER stress and antioxidant pathways

**Fig. 2:**
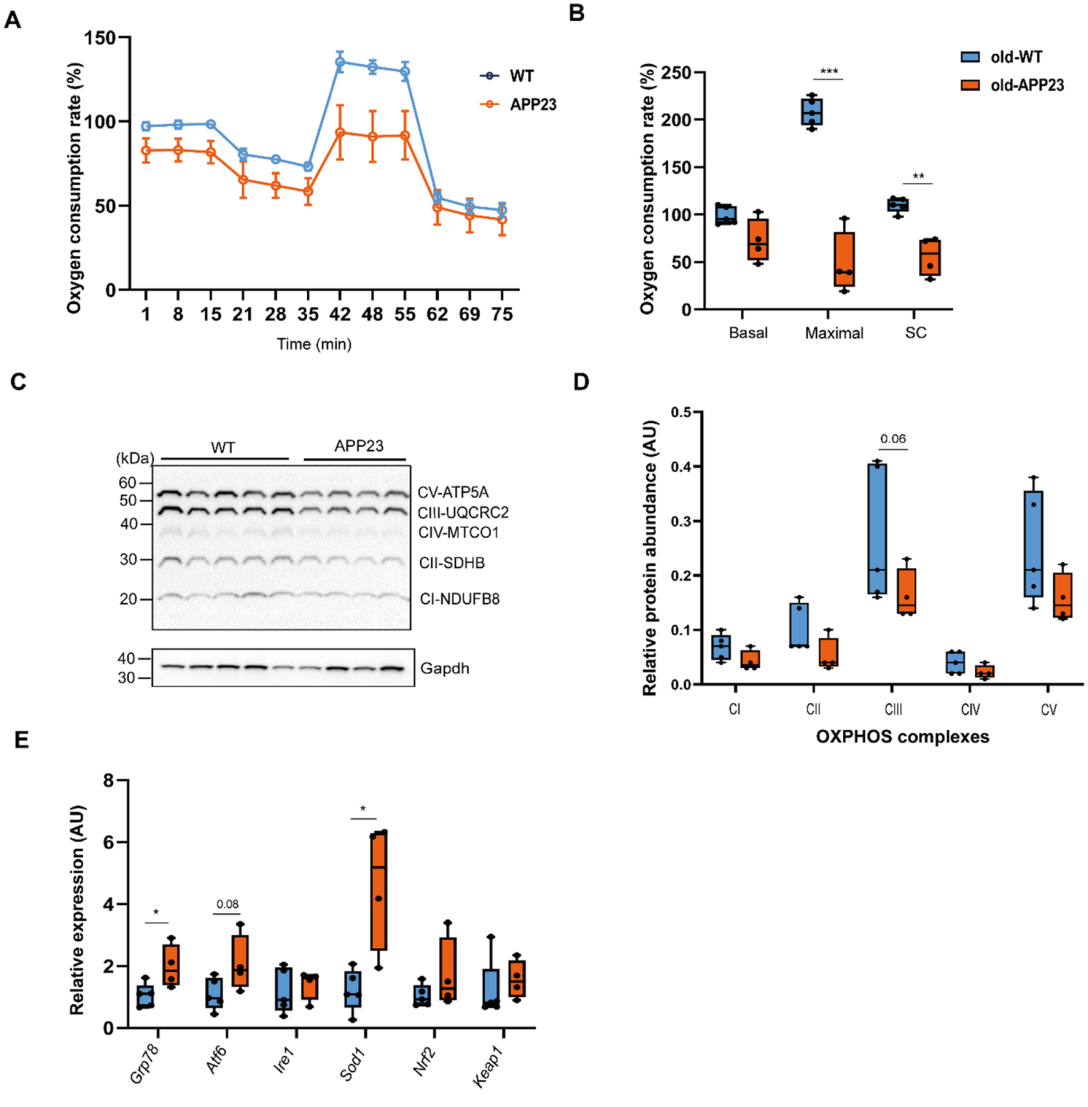
Downregulation of mitochondrial respiration in liver of 1.5-year-old APP23 mice. (**A**-**B**) Oxygen consumption rate (OCR) in primary hepatocytes of old WT and APP23 mice was calculated as the percentage of the OCR of APP23 mice relative to the baseline of the WT group and is depicted as the degree of hepatic OCR progression (**A**) and its quantification (**B**). Seahorse measurements were assessed in 2 independent experiments, each with 8 technical replicates. (**C**) Representative western blots of key OXPHOS complexes in old hepatocytes. (**D**) Quantification of OXPHOS proteins relative to GAPDH in hepatocytes. (**E**) Expression analysis of genes involved in ER stress and antioxidant effects quantified in primary hepatocytes from old mice. The data are presented as the mean ± SEM or box plots (25th to 75th percentile) with median and whiskers from minimum to maximum and were analyzed by two-way ANOVA with Bonferroni multiple comparisons test (**B**, ** D**) or independent unpaired two-tailed t test with Welch’s correction (**E**, per group). *n* = 5/4 for WT/APP23 with mixed sexes. *p ≤ 0.05, **p ≤ 0.01, ***p ≤ 0.001. AU: arbitrary units, C: complex, ER: endoplasmic reticulum, FCCP: trifluoromethoxy carbonylcyanide phenylhydrazone, OCR: oxygen consumption rate, OXPHOS: oxidative phosphorylation, R/A: rotenone + antimycin A, SC: spare capacity

In our previous study, we detected differentially regulated mitochondrial proteins in livers of APP23 females [20]. Therefore, we investigated hepatic mitochondrial respiration in 1.5-year- and 3–4-month-old mice. To evaluate mitochondrial oxidation, primary hepatocytes were isolated and their OCR measured using a Seahorse. We found a strong reduction in maximal OCR (*p* < 0.001) and a significant decrease in spare capacity (SC, *p* = 0.002) in hepatocytes from 1.5-year-old APP23 mice, whereas basal OCR was only slightly lower (F (1, 21) = 117.9, *d* = 8.259, *p* < 0.001, Fig. 2A-B). ATP turnover rates were not significantly different between old WT and APP23 mice (Supplementary Fig. 1A). We also observed a general trend towards reduced key OXPHOS complexes, particularly complex III approached significance (F (1, 40) = 0.072, *d* = 0.219, *p* = 0.06, Fig. 2C-D). Since these findings from old APP23 mice indicated impaired mitochondrial function, we hypothesized increased metabolic stress and decreased defense response in those mice. Therefore, we examined key factors of these pathways and detected the upregulation of *Grp78*, an ER stress regulator (*d* = 0.027, *p* = 0.04), and a slightly elevated *Atf6* expression (*d* = 0.054, *p* = 0.08), which activates the UPR (Fig. 2E). Furthermore, we observed significant *Sod1* upregulation in old APP23 hepatocytes compared to WT (*d* = 0.070, *p* = 0.01, Fig. 2E). Evaluating aerobic glycolysis, we observed no difference in ECAR from hepatocytes of old APP23 relative to WT mice (Supplementary Fig. 1B).

### Young APP23 do not yet indicate inflammatory signals and pathological Aß plaques in liver and brain

**Fig. 3:**
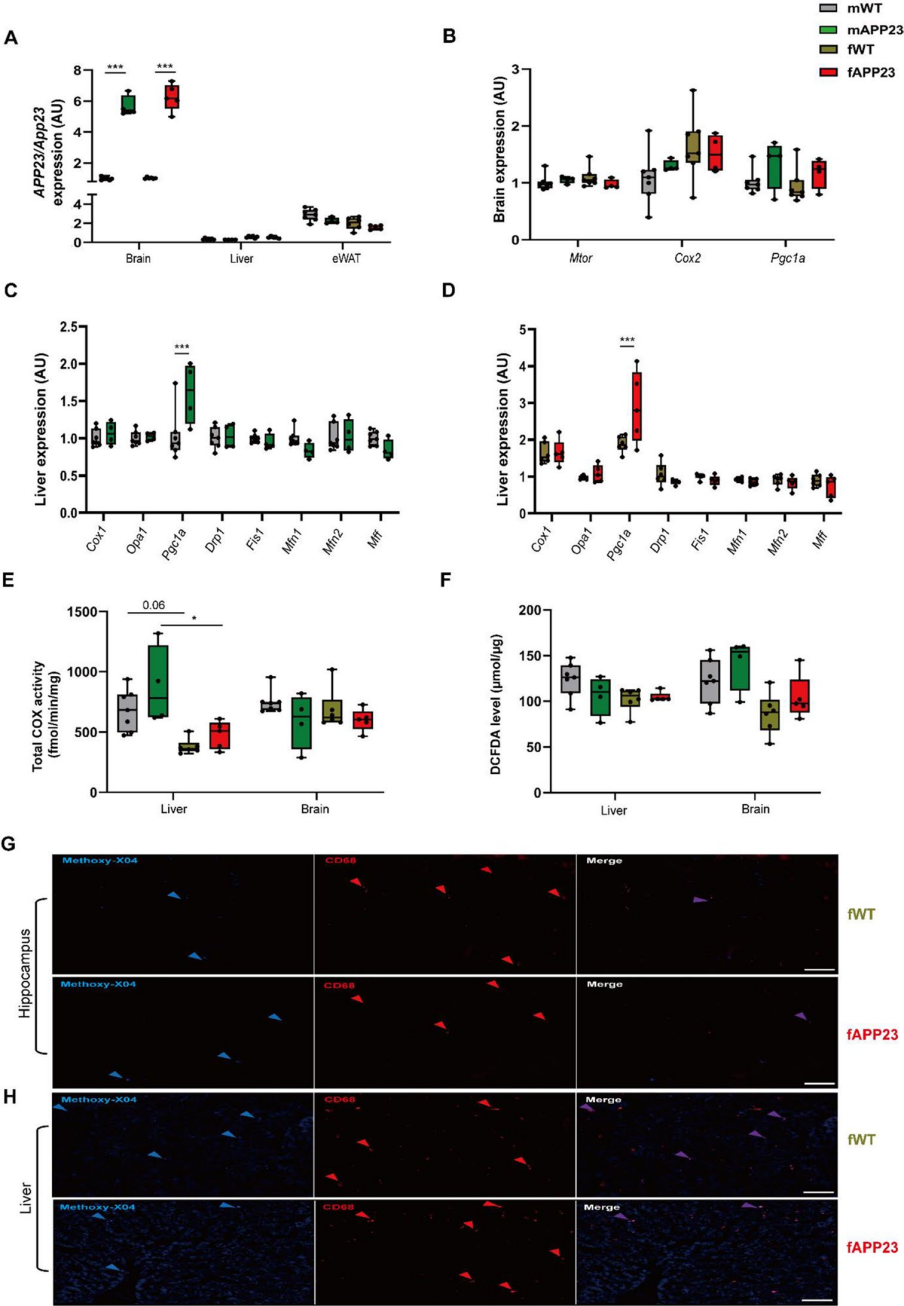
Characterization of the Aβ preplaque stage in liver and brain young APP23 mice. (**A**) Expression of the human amyloid precursor protein (*APP23*) transgene and the endogenous mouse *App* gene in brain, liver and eWAT of young male and female WT and APP23 mice normalized to *Rpl19* and all related to the male WT expression level in brain. The endogenous murine *App* transcript is detected with one mismatch (G to A) at position 5 in the fwd primer. (**B-D**) Expression analysis of genes involved in inflammation, mitochondrial biogenesis, fusion and fission quantified in brain of both sexes (**B**) as well as liver of males (**C**) and females (**D**) from WT and APP23 mice. (**E**) Total COX enzyme activity measured in liver and brain. (**F**) ROS detection in liver and brain lysates by DCFDA measurements. (**G**-**H**) IHC staining of microglia in CA1 segment of hippocampus (**G**) and macrophages in left liver lobe (**H**) by CD68^+^ and Aβ stained by methoxy-X04 in Aβ in representative sections of young WT and APP23 female mice (also refer to Supplementary Fig. 2). Data are presented as box plots (25th to 75th percentile) with median and whiskers from minimum to maximum and analyzed by three-way ANOVA with Bonferroni multiple comparisons test (**A-B**,** E-F**) or two-way ANOVA with Bonferroni multiple comparisons test (**C-D**). *n* = 7/6 for male/female WT and *n* = 4/5 for male/female APP23 for all analyses (**A-H**). *p ≤ 0.05, ***p ≤ 0.001. Scale bar: 50 μm, blue arrows indicate Aβ stained by methoxy-X04, red arrows CD68^+^ microglia/macrophages and purple arrows colocalization (**G**-**H**). Aβ: amyloid beta, AU: arbitrary units, COX: cyclooxygenase, DCFDA: 2´,7`-dichlorofluorescein diacetate, fAPP23: female APP23 transgenic mice, fWT: female WT mice, mAPP23: male APP23 transgenic mice, mWT: male WT mice, ROS: reactive oxygen and nitrogen oxide species

Since it is reported that young APP23 mice already have mild cognitive deficits [22, 24, 28], we investigated liver and brain of young APP23 mice. We analyzed inflammatory processes, COX activity, oxidative stress, mitochondrial biogenesis, and pathological Aß plaques in these organs to corroborate that 3–4-month-old APP23 mice are still in this preplaque stage. APP23 mice exhibited a strong upregulation of *APP*, the human amyloid precursor protein, in brain, but markedly less and similar levels of endogenous murine *App* in liver and eWAT (Brain: F (1, 45) = 504.4, *d* = 1.0, *p* < 0.001, Fig. 3A). However, we observed no upregulation of inflammatory response regulators in brain (Fig. 3B), but increased *Pgc1a* in liver of young APP23 males and females (Fig. 3C-D), which might suggest an induction of a compensatory response to energy deficits or in mitochondria. No inflammatory marker was dysregulated in liver or brain (Fig. 3B-D). Liver almost exclusively has COX1, with COX2 in particular being upregulated in brain of AD patients [40]. To investigate whether signs of acute inflammation were present, we also analyzed total COX enzyme activity. While total COX activity was elevated in male liver, particular in APP23 mice (F (1, 36) = 14.1, *p* = 0.02), we found no genotype-specific COX differences (Fig. 3E). COX activity in brain was comparable between all groups. Next, the inflammatory transcription factor NF-κB, which is also closely associated with COX2 in AD [41], was examined by NF-κB p50 and p65. We detect a slight increase of hepatic p50 in females, which was significantly higher in APP23 females compared to APP23 males, but no change of hepatic p65 (Supplementary Fig. 2A-B). No differences in p50 and p65 were observed in brain between WT and APP23 males and females (Supplementary Fig. 2C-D). To verify compensatory PGC1a responses, we also quantified its protein abundance, but in contrast to mRNA expression, we revealed no differences at protein level in liver or brain of young APP23 mice (Supplementary Fig. 2E-F). To assess oxidative stress, ROS and RNOS were determined in liver and brain using a DCFDA assay. Both APP23 males and females had comparable DCFDA levels in liver and brain (Fig. 3F). However, whereas liver levels were similar, females of both genotypes show lower DCFDA levels in brain. In line with this, no changes in both organs were found in young APP23 mice compared to WT performing oxiblots to detect non-enzymatic carboxylation of proteins (Supplementary Fig. 2G-H). Finally, to visualize load and size of Aβ plaques, we performed methoxy-X04/CD68 co-staining in liver and brain sections. Few and small Aβ plaques were present in the hippocampus of young mice of both genotypes (Fig. 3G and Supplementary Fig. 2I), colocalized with CD68^+^ microglia, suggesting a clearance process by phagocytosis and lysosomal degradation [40]. Consistently, similar results were obtained in liver for WT and APP23 (Fig. 3H and Supplementary Fig. 2J), in which the little amount of Aβ was colocalized with CD68^+^ macrophages [42]. In brain sections of a 1.5-year-old APP23 mouse, substantial dense-core Aβ plaques were observed in the hippocampus (Supplementary Fig. 2K), which were surrounded by CD68^+^ microglia for shielding, as these cannot by endocytosed anymore [31].

In summary, no indication of Aβ pathology or inflammatory processes could yet be detected in brain or liver of young (preplaque) APP23 mice. To investigate the metabolic capacity and systemic hepatic impact on overall health during AD pathology, we evaluated clinical liver parameters in plasma. Here, no differences of the measured parameters were recognized in old, mixed-sex mice, indicating no global liver damage in APP23 (Supplementary Fig. 3A). We also observed similar levels of those parameters analyzing plasma of preplaque male and female APP23 mice (Supplementary Fig. 3B-C). Of note, also CRP, a systemic marker for inflammation, is neither elevated in young nor old APP23 mice. This further supports our findings that there is no increased inflammation in preplaque APP23 mice.

### Preplaque APP23 females, but not males, show increased hepatic mitochondrial respiration

**Fig. 4:**
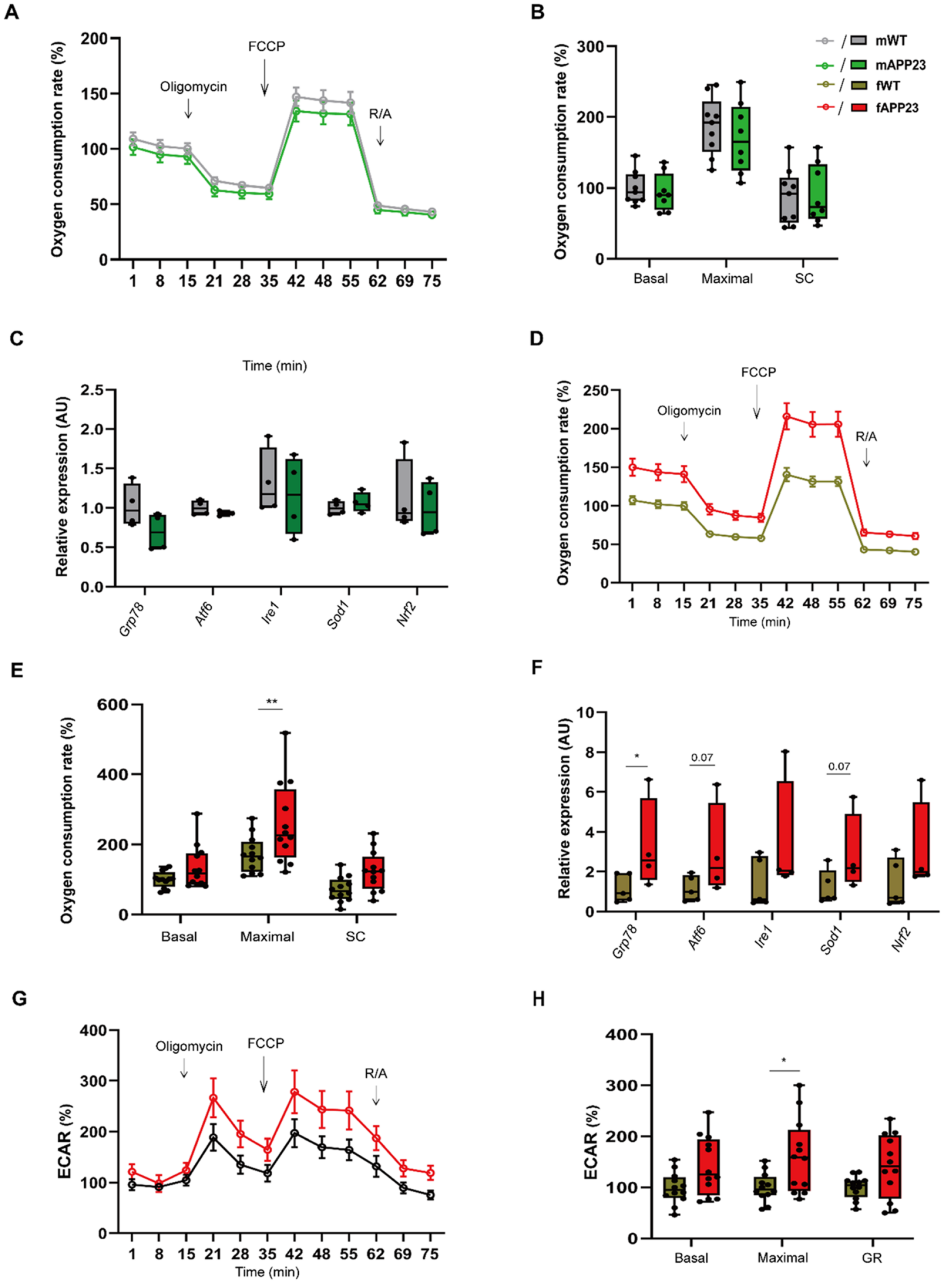
Enhanced mitochondrial respiration in primary hepatocytes of young APP23 female mice. (**A**-**B**) Hepatic OCR of young APP23 males is shown relative to the WT baseline OCRs. OCR curve (**A**) and its quantification (**B**) in primary hepatocytes from male mice. n_male_ = 9/8 for WT/APP23, measured in 3 independent experiments, each with 5 technical replicates. (**C**) Expression analysis of genes involved in ER and anti-oxidative stress response in liver of young males. n_male_ = 4/4 for WT/APP23. (**D**-**E**) Hepatic OCR (**D**) and its quantification (**E**) of primary hepatocytes isolated from young APP23 female mice. n_female_ = 12/12 for WT/APP23, measured in 4 independent experiments, each with 5 technical replicates. (**F**) Expression analysis of ER and anti-oxidative stress response in primary hepatocytes from females. n_female_ = 5/4 for WT/APP23. (**G**-**H**) ECAR curve (**G**) and its analysis (**H**) of these female hepatocytes; n_female_ = 12/12 for WT/APP23. The data are presented as the mean ± SEM or box plots (25th to 75th percentiles) with median and whiskers from minimum to maximum and were analyzed by two-way ANOVA with Bonferroni multiple comparisons test (**B**, **E**, **H**) or independent unpaired two-tailed t test with Welch’s correction (**F** per group). *p ≤ 0.05, **p ≤ 0.01. AU: arbitrary units, ECAR: extracellular acidification rate, ER: endoplasmic reticulum, FCCP: trifluoromethoxy carbonylcyanide phenylhydrazone, GR: glycolytic reserve, OCR: oxygen consumption rate, R/A: rotenone + antimycin A, SC: spare capacity

Next, we investigated hepatic mitochondrial respiration, mitohormesis and ER stress of preplaque APP23 mice to assess ATP production by mitochondrial oxidation, including potential sex differences. Primary hepatocytes were isolated from male and female mice, and their OCR was assessed during glycolytic stress. In males, the hepatic OCR did not exhibit any genotypic difference (Fig. 4A-B). Moreover, we assessed genes related to mitochondrial dynamics such as apoptosis, ER stress and anti-oxidative responses and found no evidence for an upregulation in males (Fig. 4C). In APP23 females, however, the maximal OCR was strongly enhanced (*p* = 0.004), while basal OCR and SC just missed statistical significance (F (1, 66) = 14.48, *d* = 1.623, *p* = 0.0003, Fig. 4D-E). Likewise, the ATP turnover rate tended to be increased in APP23 female hepatocytes compared to WT (*p* = 0.07, Supplementary Fig. 4A). APP23 hepatocytes also showed a higher protein concentration compared to WT cells (*p* = 0.04, Supplementary Fig. 4B), potentially indicating an increased amount of hepatic mitochondrial protein in APP23 females. Analysis of genes involved in mitochondrial oxidation, as *Ndufb8* (*d* = 0.027, *p* = 0.04), *Sdhb* (*d* = 0.04, *p* = 0.06) and *Cox7a1* (*d* = 0.007, *p* = 0.01), revealed a generally higher expression for hepatocytes isolated from preplaque APP23 females (Supplementary Fig. 4C), indicating increased mitochondrial activity. However, no difference in key OXPHOS complex I-IV abundance was observed in preplaque APP23 females (Supplementary Fig. 4D), suggesting that post-protein modifications of mitochondrial complexes may negate the effect of upregulated transcript expression. Additionally, we detected a slight overall upregulation of genes involved in ER stress responses and UPR in female hepatocytes, such as *Grp78* (*d* = 0.037, *p* = 0.05), *Atf6* (*d* = 0.047, *p* = 0.07) and *Sod1* (*d* = 0.047, *p* = 0.07), with one sample being prominent, (Fig. 4F). Furthermore, we investigated genes and proteins related to mitochondrial dynamics as potential mechanisms responding to hypermetabolic mitochondria. However, no indication of such regulatory processes was observed in female APP23 hepatocytes (Supplementary Fig. 4E). To assess whether aerobic glycolysis was affected, we examined the ECAR, which revealed a significantly higher maximal ECAR of hepatocytes from preplaque APP23 females (*p* = 0.02, Fig. 4G-H). This effect was absent in preplaque APP23 males (Supplementary Fig. 4F). There, we also found no differences in ATP turnover or proton leak (Supplementary Fig. 4G). In summary, mitochondrial respiration of APP23 females was significantly increased in the preplaque phase, a process that appears to reverse by aging under Aβ pathology.

### Preplaque APP23 females exhibit lower plasma TGs and ketone bodies, but increased NEFA release from adipocytes into circulation

**Fig. 5:**
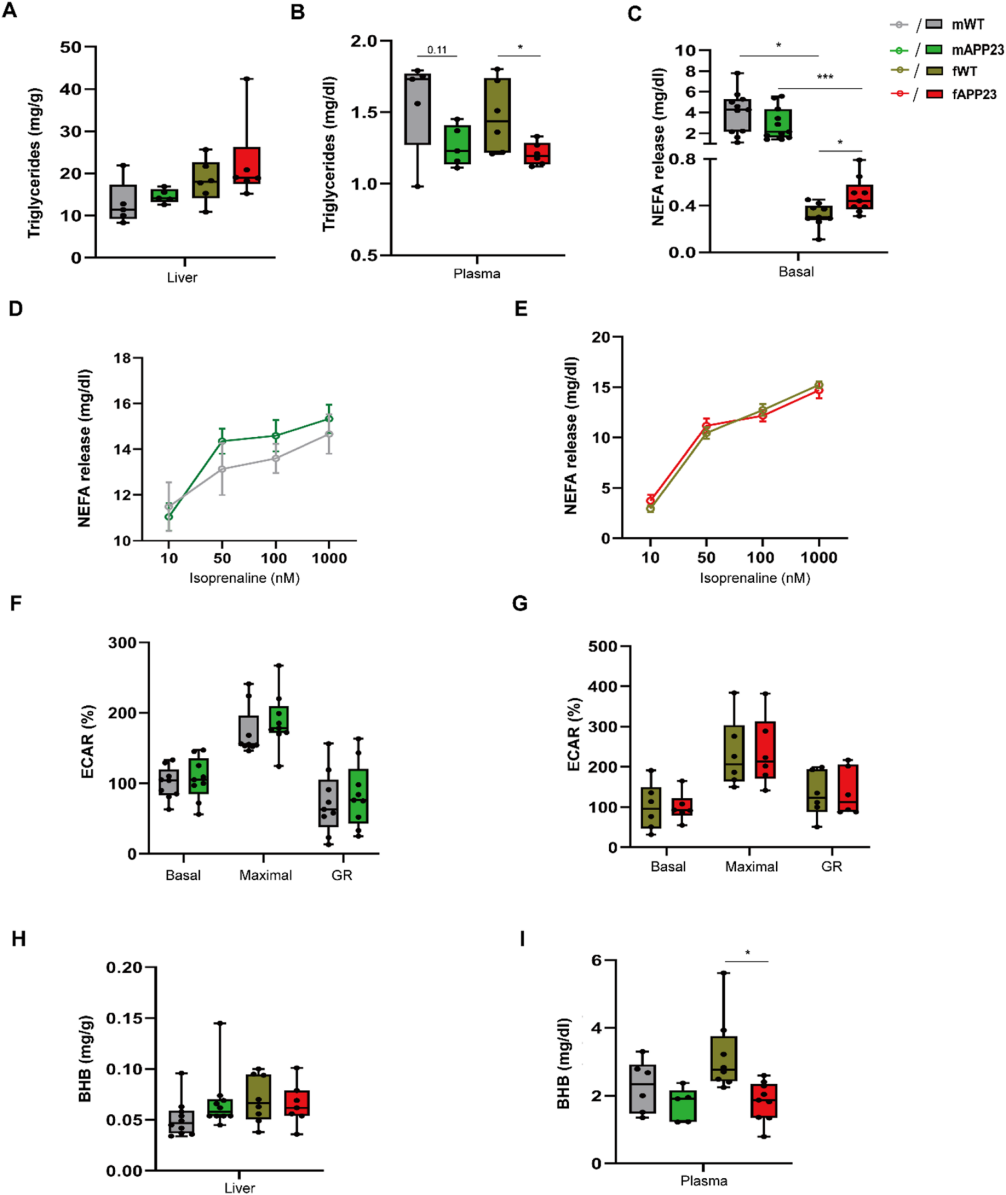
Lower circulating TG, BHB and enhanced NEFA supply in young APP23 males and females. (**A**) Hepatic TG concentrations of young male and female mice. (**B**) TG concentration in plasma samples. (**C**) Basal NEFA secretion of primary adipocytes isolated from male and female mice. (**D**-**E**) NEFA release from primary adipocytes of young males (**D**) and females (**E**) under isoprenaline stimulation ex vivo. n_male_ = 5/5, n_female_ = 6/6 for WT/APP23. (**F**-**G**) Quantification of ECAR of adipocytes differentiated from male (**F**) and female (**G**) mice in vitro. n_male_ = 9/9, n_female_ = 6/6 for WT/APP23. (**H**-**I**) BHB concentration in liver (**H**) and plasma (**I**). n_male_ = 10/10, n_female_ = 8/7 for liver, n_male_ = 6/5, n_female_ = 7/9 for plasma. The data are presented as the mean ± SEM or box plots (25th to 75th percentiles) with median and whiskers from minimum to maximum and were analyzed by Welch and Brown-Forsythe one-way ANOVA with Dunnett’s T3 multiple comparisons test between genotypes (**A**-**C**, **H**-**I**, per group) or two-way ANOVA with Bonferroni multiple comparisons test (**D**-**E**, **F**-**G**). *p ≤ 0.05, ***p ≤ 0.001. BHB: beta-hydroxybutyrate, ECAR: extracellular acidification rate, GR: glycolytic reserve, NEFA: Non-esterified fatty acid, TG: Triglyceride

We previously reported reduced hepatic TGs and a lower degree of liver steatosis in APP23 females during diet-induced obesity (DIO) [20]. Here, we evaluated circulating TG concentrations, hepatic TG storage and fatty acid release in adipocytes from preplaque mice. In liver, APP23 males and females revealed only a slight but not statistically different increase in TGs (Fig. 5A). However, plasma TGs trended to be lower in APP23 males and were decreased significantly in preplaque APP23 females (*p* = 0.035, Fig. 5B). Basal NEFA release from adipocytes was markedly reduced in females of both genotypes compared to males (F (3, 19) = 20.47, *d* = 1.407, *p* < 0.0001), whereas APP23 females showed a significantly higher NEFA secretion than WT (*p* = 0.037, Fig. 5C). As basal NEFA release was elevated in APP23 females, we also evaluated the lipolytic capacity of primary adipocytes ex vivo. Under beta-adrenergic stimulation using isoprenaline, NEFA release was similar between genotypes of the same sex (Fig. 5D-E). These findings were also in line with the ECAR measurements of ex vivo differentiated adipocytes. The acidification of medium, measured as ECAR, corresponds to NEFA release from adipocytes, as previously observed [37, 43, 44]. Thus, we evaluated ECAR of differentiated adipocytes ex vivo to measure their aerobic glycolysis. We found no evidence indicating genotype-specific effects on ECAR of differentiated adipocytes from preplaque male or female mice (Fig. 5F-G). To evaluate whether NEFAs could be used for ketone body synthesis, we also measured the BHB concentration as an estimate for ketone bodies in the liver, which did not differ between WT and APP23 mice (Fig. 5H). However, circulating BHB levels were lower in APP23 (F (3, 18) = 5.392, *d* = 0.588, *p* = 0.007), by trend in males, while APP23 females had significantly lower plasma BHB (*p* = 0.024, Fig. 5I).

### Mitochondria of SVC and adipocytes isolated from female mice are more flexible under fatty acid stress

**Fig. 6:**
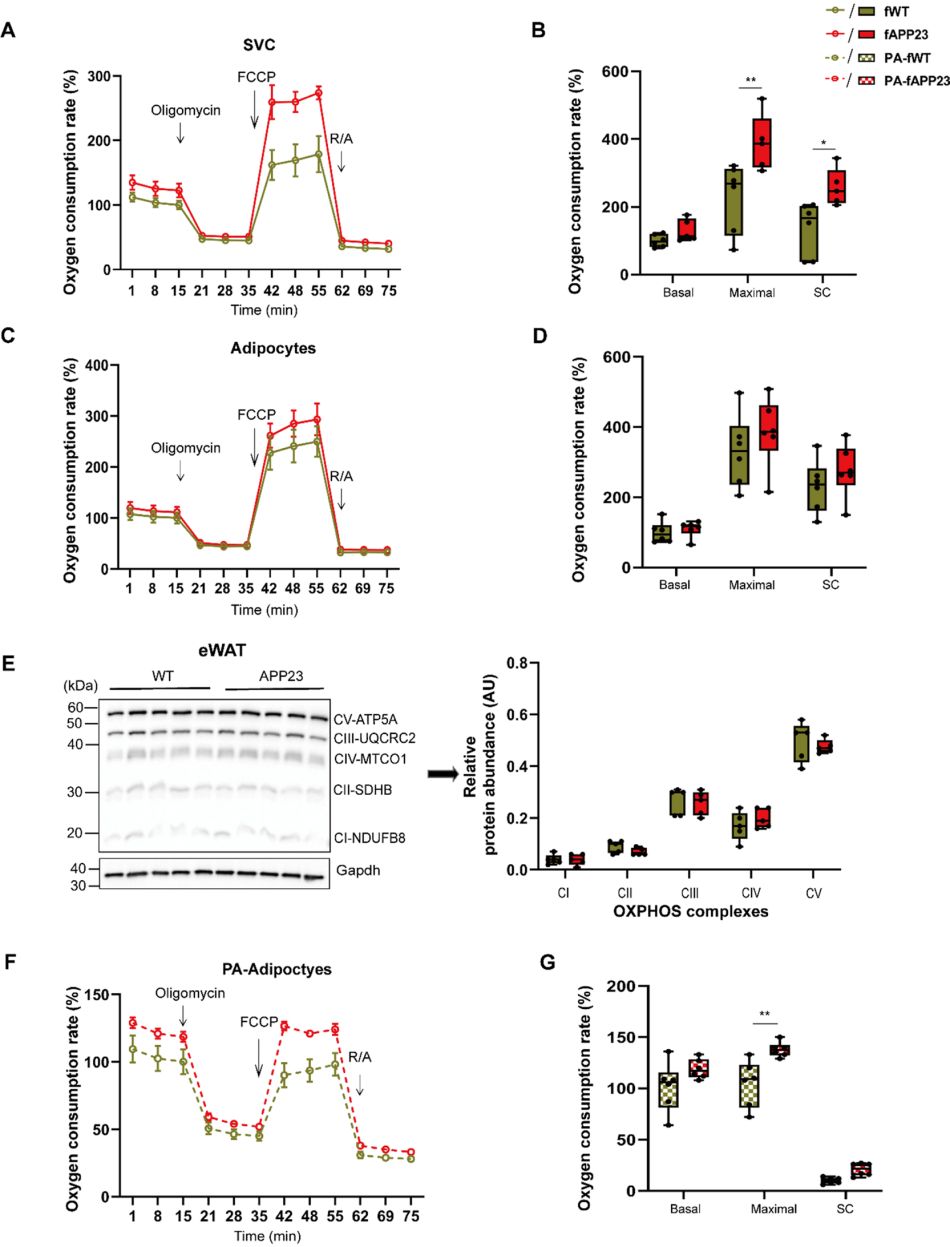
Increased mitochondrial flexibility in SVCs and differentiated adipocytes from APP23 females during fatty acid stress. (**A**-**D**, **F-G**) OCR curves (**A**, **C**, **F**) and calculations (**B**, **D**, **G**) of cultured SVCs (**A**-**B**) and differentiated adipocytes (**C**-**D**, **F-G**) derived from eWAT of young females and calculated as a percentage vs. WT. Comparative OCR analysis of SVCs (A-B, n_female_ = 6/5) and in vitro differentiated adipocytes (**C**-**D**, **F-G**, n_female_ = 6/6) under control conditions (BSA-NaCl, **C**-**D**) or fatty acid stimulation (PA, 100 µM BSA-palmitate, **F-G**). 3 independent experiments, each with 5 technical replicates. (**E**) Representative western blots of key OXPHOS complexes and corresponding quantification in eWAT of females normalized to GAPDH loading, n_female_ = 5/5. The data are presented as the mean ± SEM or box plots (25th to 75th percentiles) with median and whiskers from minimum to maximum and were ana**l**yzed by two-way ANOVA with Bonferroni multiple comparisons (**B**, **D**, **G**). *p ≤ 0.05, **p ≤ 0.01, eWAT: epigonadal white adipose tissue, FCCP: trifluoromethoxy carbonylcyanide phenylhydrazone, PA: BSA-palmitate, R/A: rotenone + antimycin A, SC: spare capacity, SVC: stromal vascular cells

To follow the reduced circulating TG and increased basal lipolytic activity of female adipocytes, we investigated mitochondrial respiration in ex vivo differentiated adipocytes. Primary SVCs, isolated from eWAT of preplaque mice, were in vitro differentiated for 10 days into were mature adipocytes and their OCR were measured with the mitochondrial stress test. We revealed an increased maximal OCR (*p* = 0.002) together with an elevated SC (*p* = 0.02) in female APP23 SVCs (F (1, 27) = 18.30, *d* = 2.706, *p* = 0.0002, Fig. 6A-B). In male SVCs, only few genotypic differences in mitochondrial respiration were detected (Supplementary Fig. 5A-B). Differentiated female adipocytes exhibited no differences in OCR between WT and APP23 (Fig. 6C-D). Accordingly, we detected a similar abundance of key OXPHOS complexes in eWAT of female mice (Fig. 6E). Likewise, differentiated male adipocytes did not differ in terms of OCR (Supplementary Fig. 5C-D).

Given that APP23 mice exhibit significantly less pronounced obesity-induced adipose tissue hypertrophy [20], potentially induced through lipid demand for hepatic ketone body production, we investigated the ability of adipocytes to respond to palmitate stimulation via mitochondrial respiration. Palmitate treatment oversupplies lipid substrates for mitochondrial oxidation, mimicking the adipose response to DIO but also provides sufficient substrates to cover the needs of APP23-derived cells. Lipid-loaded adipocytes from APP23 females (*p* = 0.002) showed a nearly 50% increase in maximal OCR (F (1, 30) = 17.80, *d* = 2.668, *p* = 0.0002, Fig. 6F-G), indicating better adaptation of mitochondrial respiration to fatty acid stress. However, this difference was much less pronounced in differentiated male adipocytes under palmitate (Supplementary Fig. 5E-F).

Finally, we examined ECAR of SVCs and differentiated adipocytes but could not detect significant sex- or genotype-specific changes in mitochondrial respiration with or without fatty acid stimulation (Fig. 5F-G, Supplementary Fig. 5G-H and Supplementary Fig. 6A-D).

### Preplaque APP23 males and females have reduced lean mass but differing activity profiles

**Fig. 7:**
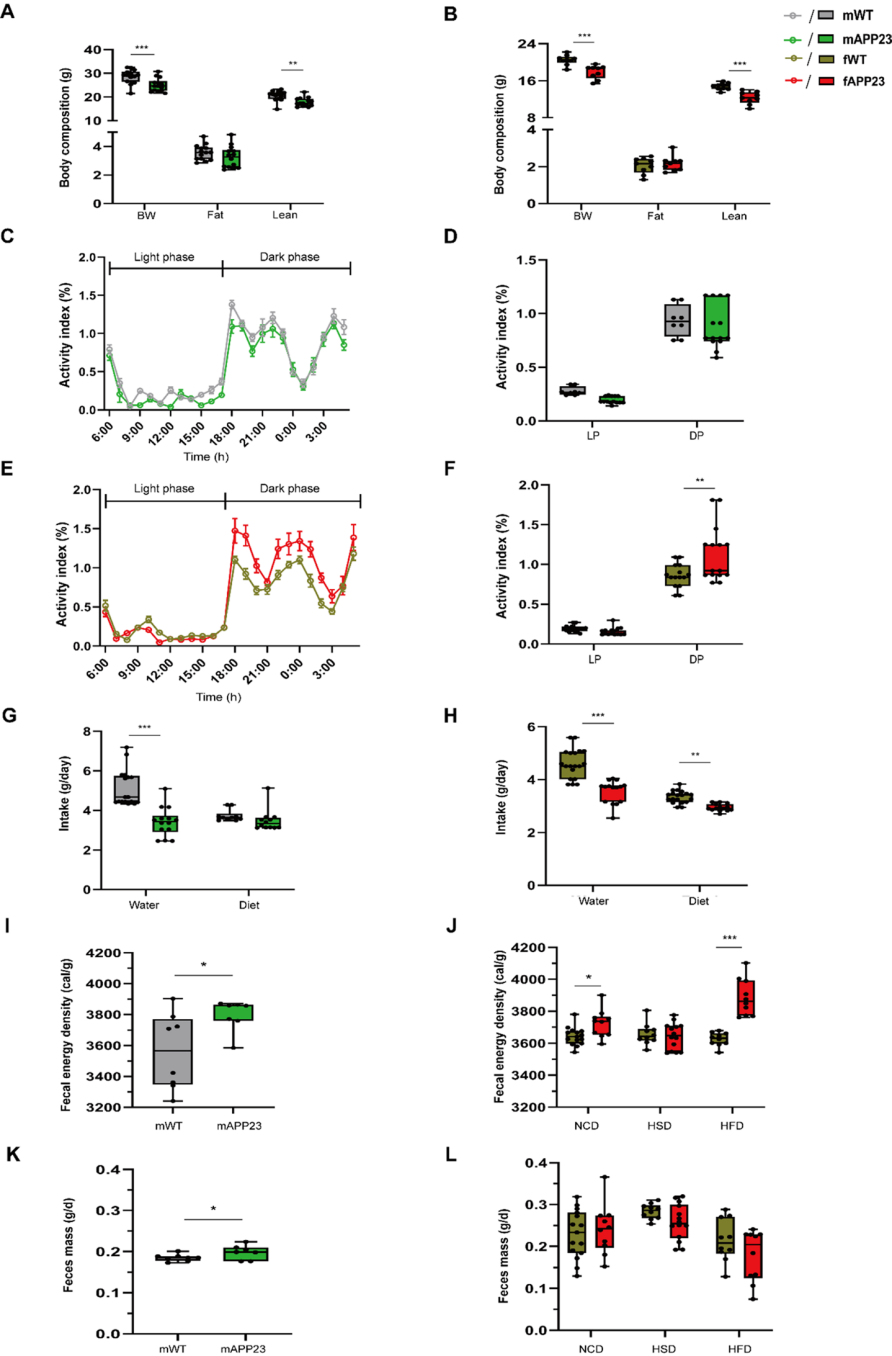
Reduced lean mass and decreased feeding with increased activity and energy loss in young APP23. (**A**-**B**) Body weight (BW), fat and lean mass of male (**A**) and female (**B**) mice. n_male_ = 14/14 and n_female_ = 9/9 for WT/APP23. (**C**-**F**) Activity profiles of male (**C**-**D**) and female (**E**-**F**) mice monitored in the DVC system as daily averages per hour (**C**, **E**) and as the means during the light (LP) and dark phases (DP) (**D**, **F**). n_male_ = 8/14 and n_female_ = 15/15. (**G**-**H**) Mean daily water and diet intake of males (**G**) and females (**H**). n_male_ = 17/14 and n_female_ = 19/15. (**I**-**L**) Mean fecal energy density (**I**-**J**) and daily feces mass (**K**-**L**) of 20-week-old males fed a 20 weeks ND (**I**, **K**) and 26-week-old females (**J**, **L**) fed 20 weeks normal-control (NCD), high-sucrose (HSD) or high-fat diet (HFD) over 48 h collection time. n_male_ = 8/7 and n_female_ = 15/10 (NCD), 10/14 (HSD), 10/10 (HFD). The data are presented as the mean ± SEM or box plots (25th to 75th percentiles) with median and whiskers from minimum to maximum and were analyzed by two-way ANOVA with Bonferroni multiple comparisons test (**A**-**B**, **D**, **F**, **G**-**H**, **J**, **L**) or unpaired two-tailed t test with Welch’s correction (**I**, **K**). *p ≤ 0.05, **p ≤ 0.01, ***p ≤ 0.001, BW: body weight, DP: dark phase, LP: light phase, NCD: normal-control diet, HFD: high-fat diet, HSD: high-sucrose diet

Given the sex-specific differences in hepatic and adipocyte mitochondrial respiration, we further analyzed the metabolic profile of preplaque APP23 males and females. We previously described reduced body weight and lean mass but increased activity in 6-week-old APP23 females [20]. Since we hypothesize that APP23 is deficient in its nutritional/energy supply, we investigated growth during early development. This confirmed our previous results in APP23 females and showed that young APP23 males also had lower body weight and lean mass, but not fat mass (all *p* ≤ 0.002, Fig. 7A-B). Male (*p* = 0.13) and female (*p* = 0.09) APP23 mice showed slight indication of reduced body length (F (3, 41) = 3.22, *d* = 0.046, *p* = 0.03, Supplementary Fig. 6E), but no general, serious growth defects were observed at the preplaque stage. To investigate whether APP23 males also show enhanced activity, we continuously monitored the cage activity of preplaque male and female mice, separated by genotype in the DVC system, and calculated their activity profiles (Fig. 7C, E). These activity data were comparable to those previously obtained with single-housed mice monitored in a TSE PhenoMaster. We were able to verify the activity phenotype of preplaque APP23 females on ND, which was elevated by 20% during dark phase (*p* = 0.004), whereas young APP23 males exhibited a similar activity profile to that of WT (females: F (1, 56) = 5.671, *d* = 1.049, *p* = 0.02, Fig. 7C-F). Furthermore, water intake was reduced in preplaque male and female APP23 (all *p* < 0.001), and APP23 females also showed a decreased diet consumption of approximately 15%, while males fed almost 10% less, but missed significance (Fig. 7G-H). However, not only was the calorie intake of APP23 mice altered, but also the amount of energy lost via the feces. To evaluate fecal energy loss, mouse feces were collected from single-house mice over 48 h during indirect calorimetry at an age of 20 weeks for males and 27 weeks for females after completion of different dietary interventions, and the fecal energy density was measured by combustion calorimetry. Fecal energy density was increased in APP23 males fed with normal-control diet (NCD, *p* = 0.03, Fig. 7I). Females lost more energy via feces under an NCD (*p* = 0.04, Fig. 7J). This effect was not observed on high-sucrose diet (HSD), but was evident with a high-fat diet (HFD, *p* < 0.001). Additionally, APP23 males produced slightly more feces (*p* = 0.03), which increased their total energy loss (Fig. 7K). While females generally produced more feces than males, we detected no genotype-specific differences in feces mass in terms of diet (Fig. 7L).

### APP23 mice exhibit increased premature mortality risk and distinct feeding and activity patterns shortly before death

**Fig. 8:**
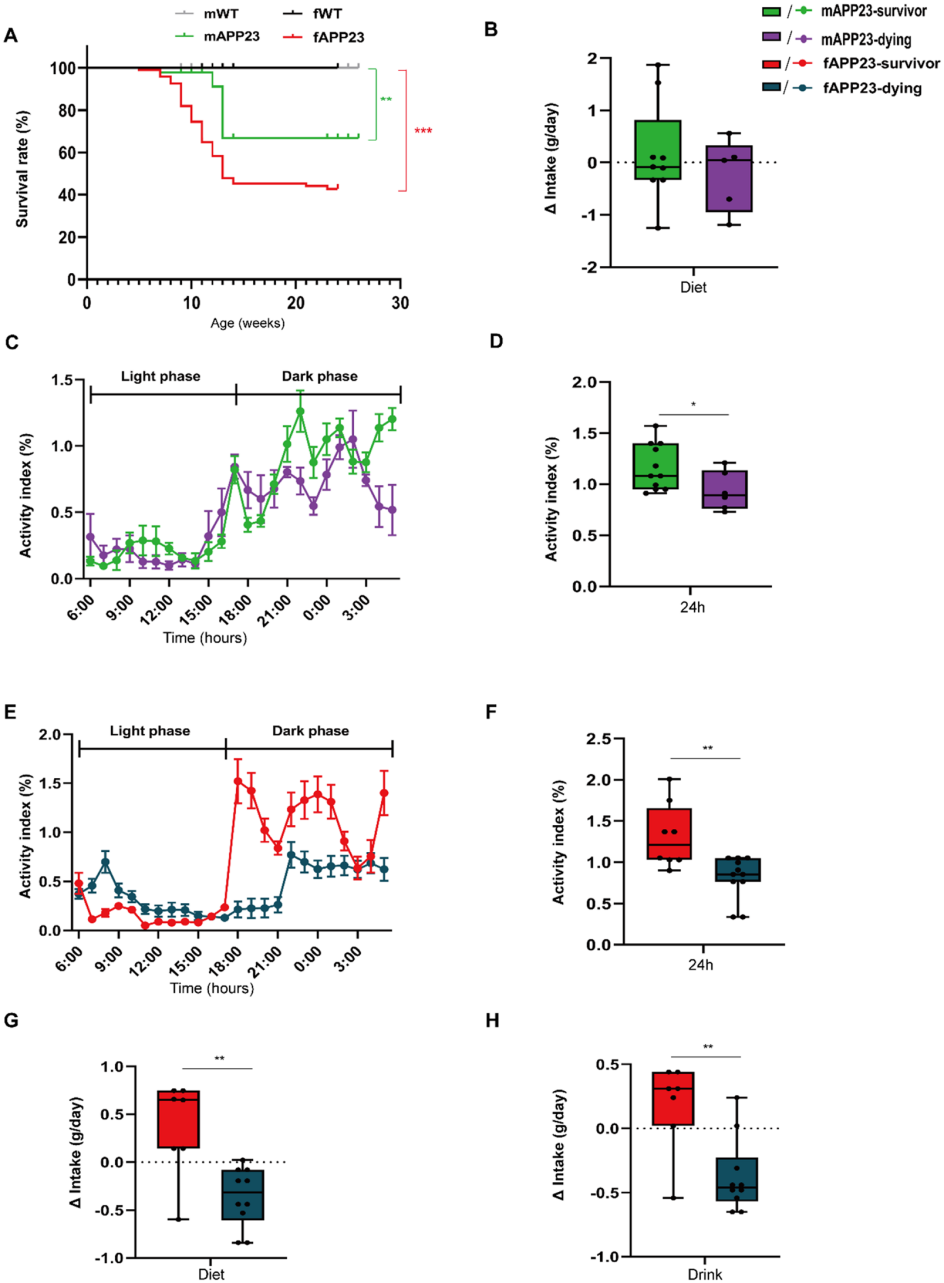
Dying APP23 females exhibit reduced activity and further decreased diet intake before death. (**A**) Sex- and genotype-specific survival rates of WT and APP23 mice. (**B**, **G**) Changes in the diet intake of APP23 males (**B**) and females (**G**) 10 days before death. (**C**-**F**) 24 h activity of APP23 males (**C**, **D**) and females (**E**, **F**). (**H**) Drink intake of APP23 females. Number of surviving/dying mice: n_female_ = 8/11 and n_male_ = 11/6 for activity, n_female_ = 7/10 and n_male_ = 9/5 for diet, n_female_ = 7/10 for drink. The data are presented as Kaplan‒Meier curves, mean ± SEM or box plots (25th to 75th percentiles) with median and whiskers from minimum to maximum and were analyzed by log-rank tests (**A**) and unpaired two-tailed t tests with Welch’s correction (**B**, **D**, **F**-**H**). *p ≤ 0.05, **p ≤ 0.01, ***p ≤ 0.001

We and others observed increased mortality in APP23 mice [29], particularly during the preplaque stage (Fig. 8A). Hence, we analyzed survival of male and female APP23 mice to reveal sex- and genotype-specific differences. We detected an increased mortality rate, in APP23 males (*p* = 0.008) and females (*p* < 0.001), with more than 50% of APP23 female mice dying during throughout the analysis (Fig. 8A). Notably, mortality of APP23 females was 3.4-fold higher than that of males (*p* < 0.001, Table 1).

**Table 1:** Survival rates of male and female WT and APP23 mice until sacrifice

	Male		Female	
	WT	APP23	WT	APP23
n, total	41	45	56	94
n (%) of mice, dead	0 (0%)	7 (16%)	0 (0%)	51 (54%)
n (%) of mice, survived	41 (100%)	38 (84%)	56 (100%)	43 (46%)
Mean age at death, weeks	n/a	12	n/a	11

Since mice were continuously monitored, we retrospectively evaluated their data 10 days before death by grouping the surviving and dying APP23 mice. Diet intake of dying APP23 males did not change, but their activity decreased (*d* = 0.015, *p* = 0.03, Fig. 8B-D). Activity of APP23 females, which otherwise exhibit increased activity, decreased by more than 60% before death (*d* = 0.005, *p* < 0.01, Fig. 8E-F). Furthermore, in contrast to males, dying APP23 females further reduced their diet consumption (*p* = 0.005), whereas survivors enhanced it (Fig. 8G), and this was also observed for water intake (*p* = 0.008, Fig. 8H).

## Discussion

Previously, we reported that APP23 females fed an NCD exhibit differential regulation of mitochondrial and energy pathways before Aβ plaque formation [20]. Here, we further characterized early metabolic profiles and cellular energy alterations to gain novel insights into the APP23 mouse model for AD. Old APP23 mice exhibited decreased mitochondrial respiration accompanied by the upregulation of ER stress markers and antioxidant genes. ER stress induced by Aβ and tau deposition activates adaptive UPR pathways initially, but chronic activation can eventually exacerbate pathology and contribute to cellular damage and death [45, 46]. However, an important caveat interpreting these data is the “survivor effect”, as our analysis of 1.5-year-old APP23 mice inherently includes only those APP23 mice that lived until the experiment. We controlled for aging per se by also analyzing matching WT littermates, but for the old APP23 it remains unclear whether the reduced mitochondrial respiration may have contributed to their survival. Therefore, when interpreting age-related mitochondrial changes, it should be considered that selection of old APP23 mice that survived until analysis may be a potential confounding factor.

In line with previous research, we found no indication of inflammatory processes or oxidative stress during the preplaque stage [47–49]. Young APP23 mice already display mild cognitive deficits prior to significant Aβ deposition [22, 24, 28]. In young APP23, we only detected few and small Aβ plaques in brain and liver. These were colocalized with microglia or macrophages indicating ongoing Aβ clearance by lysosomal degradation [50, 51]. This aligns with reports suggesting that substantial Aβ plaques formation starts around 6 months of age [21, 52]. Early spatial memory deficits preceded increased accumulation of Aβ (1-42) peptides and plaque formation in young APP23 mice [28]. Likewise, no difference in Aβ plaque load, pro-inflammatory cytokines and hippocampal microglia number was observed during the preplaque stage in APP23, but were present in transgenic mice at 1 year of age [52]. As disease progresses, Aβ accumulation becomes excessive, resulting in dense-core plaques formation that is encapsulated by glial cells [53, 54], which are then shielded by microglia or macrophages to reduce further growth as shown in old APP23 mice [31]. Aβ is naturally produced in peripheral tissues such as eWAT and liver, in which we revealed low but endogenous *App* expression, and accumulates both peripherally and centrally, where clearance mechanisms progressively decline in AD [34–36, 40, 42]. Thus, hepatic accumulation of Aβ could also affect the liver function, contributing to further impairment of systemic metabolism.

An early upregulation of hepatic mitochondrial respiration in young APP23 females could reflect compensatory responses to increased synaptic mitochondrial energy demands, observed in early AD stages [55, 56]. These compensatory mechanisms might paradoxically lead to metabolic exhaustion, contributing to premature mortality. This interpretation aligns with evidence from AD mouse models; wherein early hyperactive mitochondria lead subsequently to excessive ROS generation and synaptic dysfunction [17]. Thus, metabolic changes identified in younger animals could serve as early indicators of AD onset, yet caution is required in drawing definitive conclusions.

We also investigated lipid parameters and found decreased circulating NEFA and TG in young APP23 females. Since plasma TG remained reduced despite enhanced adipocyte NEFA release, this may indicate higher utilization of fatty acids as substrates for mitochondrial β-oxidation rather than TG synthesis or storage. Moreover, adipocytes from APP23 females displayed improved mitochondrial flexibility under fatty acid stress, suggesting adaptive responses to high-energy demands [57–59]. BHB is one of the most abundant circulating ketone bodies [60], approximately 70% of which originates from liver [61] and can cross the blood-brain barrier [62]. The lower BHB levels in APP23 females might reflect increased cerebral utilization to compensate for reduced glucose availability, thus sustaining brain function and synaptic energy supply. Ketogenesis is initiated when glycogen stores are depleted, leading to NEFA release from adipocytes, which was observed in APP2 females. Since ketone bodies are not stored in the liver but rapidly released into the bloodstream, lower plasma levels of ketones might indicate increased consumption, primarily by the brain. Although the brain’s primary energy source is glucose, ketone bodies can support synaptic transmission, particularly by maintaining ion gradients [63, 64]. Thus, the decreased plasma BHB in APP23 females may suggest a utilization of it as alternative fuel for the brain, compensating for reduced glucose availability. In this context, the reduced BHB plasma levels in female APP23 might potentially contribute to premature death, together with reduced food intake, as the energy requirements could finally no longer be maintained. However, APP23 males showed a partially different phenotype, with elevated fecal energy loss (calories and mass) and lower body weight without an increase in activity, contributing to chronic negative energy balance and higher mortality. Similarly, decreased mitochondrial respiration in the brain was observed in 1 to 4-month-old AD mice [56, 65, 66] and increased lipid peroxidation in the brain of 3-month-old AD mice [67]. Brain mitochondrial dysfunction and peripheral ketone compensation are known to precede early-stage AD [15]. The variation in circulating BHB between male and female APP23 mice may reflect sex-specific differences in brain fuel utilization. This hypothesis should be investigated in future studies.

Consistent with other studies, lower body weight and reduced lean mass were observed in both female and male APP23 mice [68–70] and in AD patients, indicating that weight loss implies the progression of mild cognitive impairment [71]. The loss of lean mass likely results from muscle degradation and malnutrition. This also suggests that APP23 mice are prone to accelerated aging [72]. In agreement with previous findings, enhanced activity in AD models has already been described [73–75]. We hypothesize that the combination of higher energy expenditure, lower caloric intake and elevated fecal energy loss contributes to premature death of APP23 mice. Survival analysis revealed greater mortality in APP23 mice within a very specific time frame, with females being particularly affected, as corroborated by others [29]. Dying females decreased their activity and intake, possibly as a protective mechanism to conserve energy or as a sign of fatal emaciation [76]. The increased mortality may be associated with this chronic energy deficit. Indeed, metabolic dysfunction and mitochondrial insufficiency are established contributors to seizure activity and sudden death in other AD models, such as APP/PS, of which about 25% die [77]. Since seizure are closely linked to insufficient cerebral energy supply and mitochondria dysfunction [78], this link emphasizes the importance of understanding bioenergetic regulation in the early progression of AD. Thus, we highlighted that early alterations in mitochondrial respiration in young APP23 females might be an indicator of AD onset. In line with our previous report, alterations in hepatic mitochondrial oxidative proteins and energy pathways in APP23 females occurred before Aβ plaque formation [20]. Our findings revealed elevated mitochondrial flexibility in young females, enhancing the oxidative response to high energy demand. This suggests increased substrate oxidation (glucose, fatty acids) for ATP production to meet energy needs. This compensatory mechanism, potentially arising from brain glucose hypometabolism and synaptic mitochondrial dysfunction, may lead to overcompensation of the energy supply in young APP23 females. However, this excess becomes depleted in 1.5-year-old APP23 over time, resulting in the observed loss of mitochondrial respiration capacity. AD is a highly complex disease and it is likely that the aging-related metabolic decline results from the interaction of several mechanisms, involving various brain cells, hepatocytes, adipocytes, and myocytes. Previous studies reported hyperactive mitochondria and excessive ROS in neurons of 3-month-old APP23 mice, which contributed to later synaptic loss [17]. We hypothesize that early changes in APP23 mice, such as hyperactivity, negative energy supply and mitochondrial dysfunction, may drive upregulation of hepatic mitochondrial respiration. Future research should investigate the potential mechanism of hepatic oxidative stress and ROS regulation in APP23 mice and AD, with particular emphasis on sex differences.

These differences between APP23 females and males might be related to hormonal effects affecting satiety/appetite regulation. Unlike females, young males exhibited no strong alterations in hepatic or adipose mitochondrial oxidation or metabolic phenotypes. These sex-specific energy derangements are likely driven by hormone disparities, particularly estrogen and testosterone. Estrogen influences appetite signals, regulates satiety hormones, boosts metabolic rates and energy expenditure, and enhances fat utilization through beta-adrenergic signaling [79]. This might also entail sex-related effects in terms of a beta-adrenergic signaling-mediated stress response. Modulation of the HPA axis by estrogen may be due to changes in glucocorticoid receptor-mediated negative feedback mechanisms [80]. We suspect that estrogen could be a key factor for the effects in APP23 females. Estrogen regulates leptin release from adipocytes via hypothalamic receptor modulation, suppresses appetite and increases energy expenditure in females [81]. It may also increase fat utilization through beta-adrenergic signaling. Yet, the effects of estrogen on food consumption are complex and depend on factors such as genetics, lifestyle and hormonal balance.

Moreover, previous studies have shown sex differences in the Aβ burden of APP/PS1 mice, with female mice displaying higher levels of Aβ [82]. Hence, Aβ burden might exhibit an influence on the observed energy differences between sexes. This is for example corroborated by the observation of lower weight with decreased adiposity and increased energy expenditure in 3-month-old Tg2576 mice prior Aβ plaque deposition [83]. This study suggests that excess Aβ can disrupt hypothalamic arcuate neuropeptide Y neurons leading to weight loss and a pathologically low leptin state in early disease development. However, further investigations are warranted to dissect the underlying mechanisms of premature mortality risk and differences between male and female mice in the preplaque stage. It should be borne in mind that mice are used at 6–9 months of age at the earliest for AD-related research on the APP23 mouse model, as these mice are already preselected by their phenotype/survival.

Limitations of our work include the small sample size of 1.5-year-old APP23 mice due to elevated mortality that precluded comprehensive and sex-specific analysis of mitochondrial respiration experiments. The increased mortality is a selective confounder allowing only age-related investigation of surviving APP23 mice, whose mitochondrial function may be altered specifically because of this survival advantage. Furthermore, our results are solely based on the APP23 model and may differ in other transgenic AD models, as highlighted by sex-specific metabolic alterations in models like triple-transgenic AD or E4FAD (human APOE4 and five familial AD mutations) females [84, 85]. Due to the complexity of analyzing sex differences as independent characteristics of humans, we did not conduct an in-depth analysis focusing on independent factors, as such requires separate studies [86]. Moreover, multiple factors, including seizures tendency, psychological factors, cardiovascular health, gastrointestinal absorption, and many more should also be considered for the increased mortality of young APP23 mice. Future studies are warranted to identify predictive biomarkers of mortality in these AD mouse models.

### Perspectives and significance

Sex differences significantly influence AD progression, yet their impact on energy metabolism remains understudied. Our findings offer novel perspectives into the early metabolic changes preceding pathological Aβ plaque formation, highlighting mitochondrial dysfunction, energy imbalance, and sex-specific metabolic differences in the early onset of Alzheimer’s disease (AD). The observed hyperactive mitochondrial respiration in preplaque APP23 females suggests a compensatory but energy depleting mechanism resulting in an increasing mortality risk. Our results emphasize the importance of systemic metabolic regulation in AD pathology and underscore the necessity of future mechanistic studies focused on sex-specific metabolic differences, hormonal influences, and interactions between peripheral tissues and neuronal energy metabolism.

## Conclusion

In conclusion, we observed virtually no AD-related inflammatory processes and pathological Aβ plaque deposition, still under clearance by phagocytes in brain and liver of young APP23 mice, which indicates an AD preplaque stage. However, these early metabolic alterations, including altered mitochondrial respiration and systemic energy imbalance, are sex-specific and may predispose to premature mortality. Future research is warranted to explore the underlying mechanisms driving these sex-specific metabolic differences and investigate the interactions between various cells and tissues contributing to these effects.

## Electronic supplementary material

Below is the link to the electronic supplementary material.

Supplementary Material 1

## Data Availability

Data is provided within the manuscript or supplementary information files and will be made available by the corresponding author upon reasonable request.

## Abbreviations

3xTg-AD: Triple-transgenic AD mice
AD: Alzheimer’s disease
Aβ: Amyloid-β
Atf6: Activating transcription factor 6
AU: Arbitrary units
BHB: Beta-hydroxybutyrate
BW: Body weight
C: Complex
Cox7a1: Cytochrome c oxidase subunit 7A1
DCFDA: 2´,7`-dichlorofluorescein diacetate
DIO: Diet-induced obesity
DVC: Digital Ventilated Cage
DP: Dark phase
E4FAD: human APOE4 and five familial AD mutations mouse model
ECAR: Extracellular acidification rate
ER: Endoplasmic reticulum
eWAT: Epigonadal adipose tissue
f: Female
Gpr78: G protein-coupled receptor 78
GR: Glycolytic reserve
HFD: High-fat diet
HPA axis: Hypothalamic–pituitary–adrenal axis
HSD: High-sucrose diet
Ire1: Serine/threonine-protein kinase and endoribonuclease
LP: Light phase
m: Male
NCD: Normal-control diet
ND: Normal diet
NEFA: Non-esterified fatty acids
NMR: ^1^H-magnetic resonance spectroscopy
Ndufb8: NADH: ubiquinone oxidoreductase subunit B8
OCR: Oxygen consumption rate
OXPHOS: Oxidative phosphorylation
PA: BSA-Palmitate
Ppia: Peptidylprolyl isomerase A
ROS: Reactive oxidation species
SC: Spare capacity
Sdhb: Succinate dehydrogenase complex, subunit B, iron sulfur
Sod1: Superoxide dismutase 1
SVCs: Stromal vascular cells
TG: Triglyceride
UPR: Unfolded protein response
y: Young
