# Supplementary material for: Sex-specific changes in energy demand during the preplaque stage in a transgenic Alzheimer’s mouse model
Source: Biol Sex Differ. 2025 Jul 17;16:54. doi: 10.1186/s13293-025-00737-0 (PMC12273039; doi:10.1186/s13293-025-00737-0)
Supplement: Supplementary file 1 — Supplementary Material 1 [file 13293_2025_737_MOESM1_ESM.docx]

**Sex-specific changes in energy demand during the preplaque stage of a transgenic Alzheimer’s mouse model**

Rongwan Sun, Leonie-Kim Zimbalski, Stefanie Schreyer, David Baidoe-Ansah, Aida Harutyunyan, Arnd Heuser, Rachel N Lippert, Joachim Spranger, Knut Mai, Sebastian Brachs

**Supplementary Figures**

**
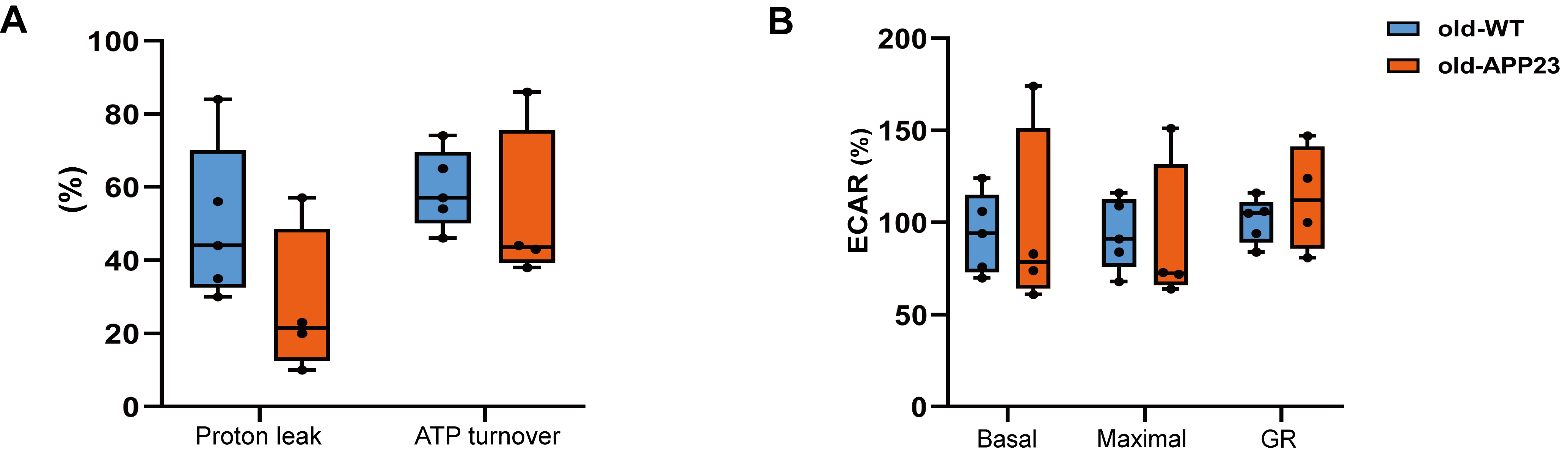
**

**Supplementary Figure 1. (A-B)** Proton leak and ATP turnover (**A**) as well as ECAR (**B**) evaluated in primary hepatocytes from 1.5-year-old mixed-sex WT and APP23 mice. n = 5/4 for WT/APP23 with mixed sexes. The data are presented as box plots (25^th^ to 75^th^ percentile) with median and whiskers from minimum to maximum and analyzed by two-way ANOVA with Bonferroni multiple comparisons test.


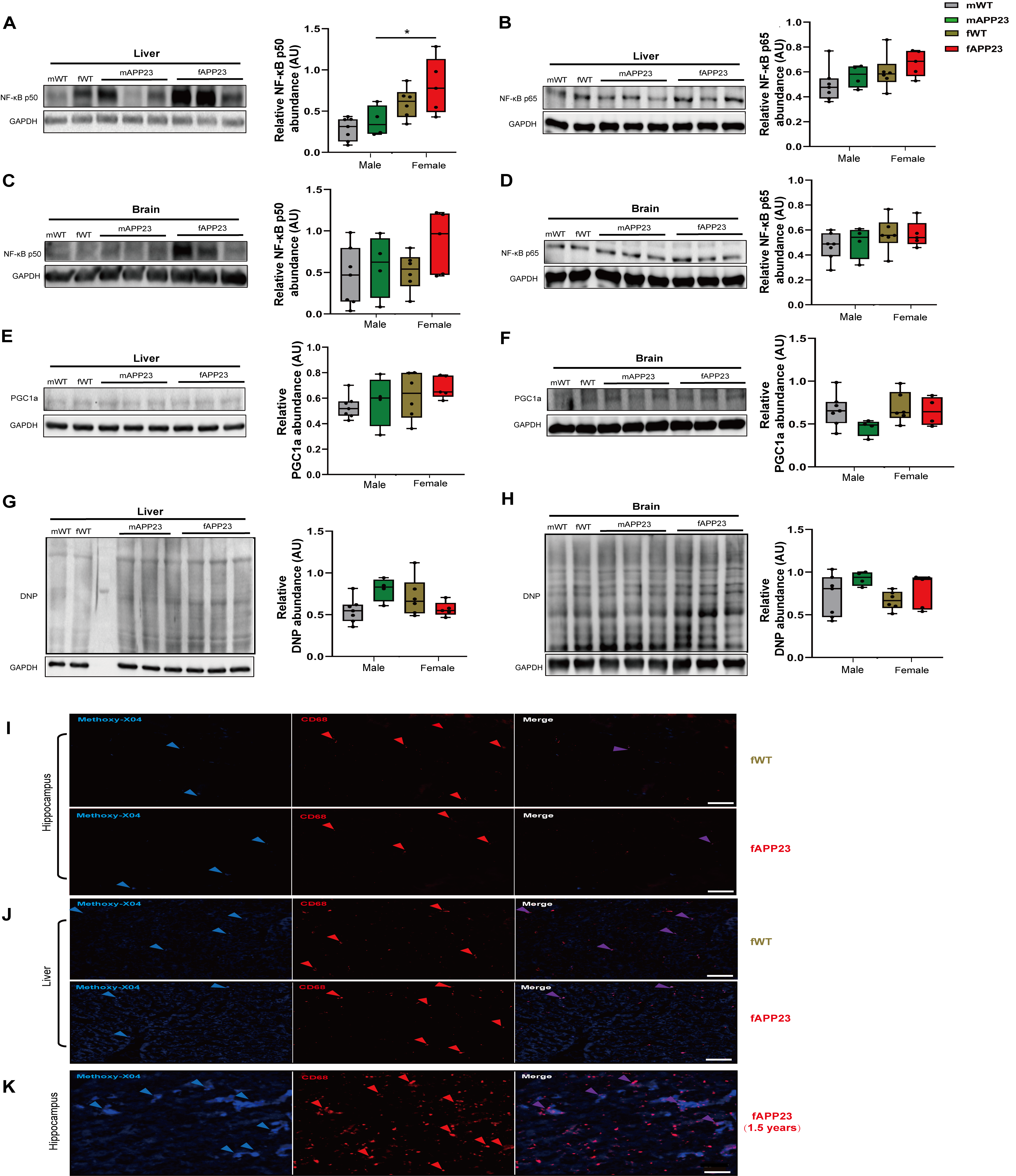


**Supplementary Figure 2.** **Aβ preplaque stage characterization in liver and brain of mixed-sex young APP23 mice.** (**A-C**) Representative western blots and their quantification of NF-κB p50 (**A, C**) and p65 (**B, D**) normalized to GAPDH loading control from liver (**A-B**) and brain (**C-D**) lysates of young male and female WT and APP23 mice. (**E-F**) Representative western blots and corresponding protein quantification of PGC1a in liver (**E**) and brain (**F**). (**G-H**) Oxiblots using anti-DNP antibody for detection of protein carboxylation to estimate ROS in liver (**G**) and brain (**H**) lysates. (**I-K**) Representative IHC staining of microglia in CA1 segment of hippocampus (**I**) and macrophages in left liver lobe (**J**) by CD68^+^ and Aβ staining with methoxy-X04 in representative sections of other young female WT and APP23 mice and hippocampus of 1.5-year-old APP23 mouse (**K**) as positive control (referring to Figure 3). n = 7/6 for male/female WT and n = 4/5 for male/female APP23 for all analyses. Data are presented as Data are presented as box plots (25^th^ to 75^th^ percentile) with median and whiskers from minimum to maximum and analyzed by one-way ANOVA with Bonferroni multiple comparisons test (**A**-**H**). *p ≤ 0.0Scale bar: 50 μm, blue arrows indicate Aβ stained by methoxy-X04, red arrows CD68^+^ microglia/macrophages and purple arrows colocalization. Aβ: amyloid-β, AU: arbitrary units, DNP: dinitrophenylhydrazone, ROS: reactive oxygen species.


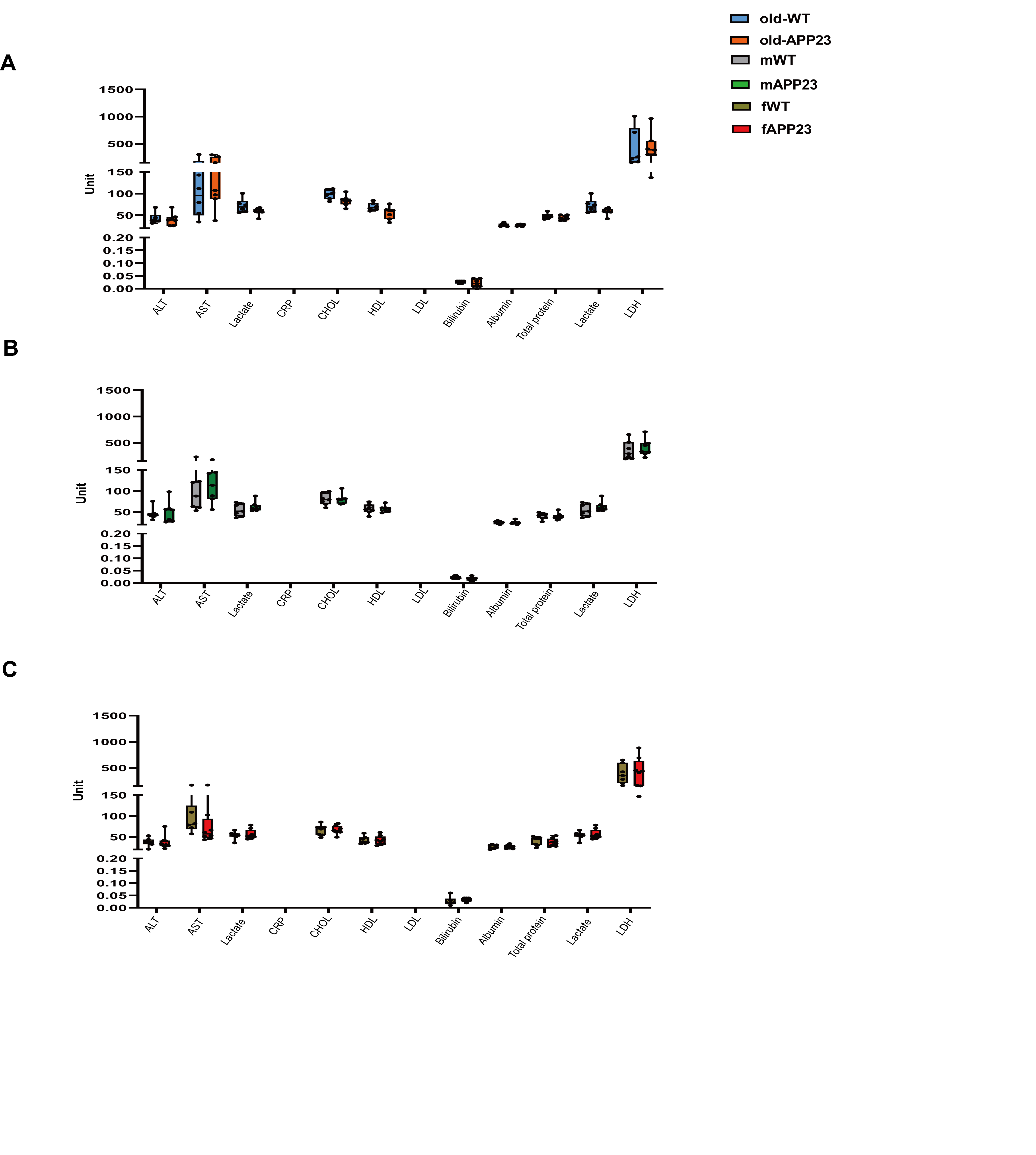
**Supplementary Figure 3. Hepatic parameters in plasma for function and overall health.** (**A**-**C**) Liver parameters measurement in plasma of 1.5-year-old mixed-sex mice (**A**) or young females **(B**) and males (**C**), including assessment of liver function impairment, liver total protein synthesis, bile acid and lactate levels, and inflammatory response. n_old_ = 6/7 for mixed-sex, n_male_ = 7/8 and n_female_ = 7/7 for WT/APP23. Data are presented as box plots (25^th^ to 75^th^ percentile) with median and whiskers from minimum to maximum and analyzed by two-way ANOVA with Bonferroni multiple comparisons test (**A**-**C)**.

**
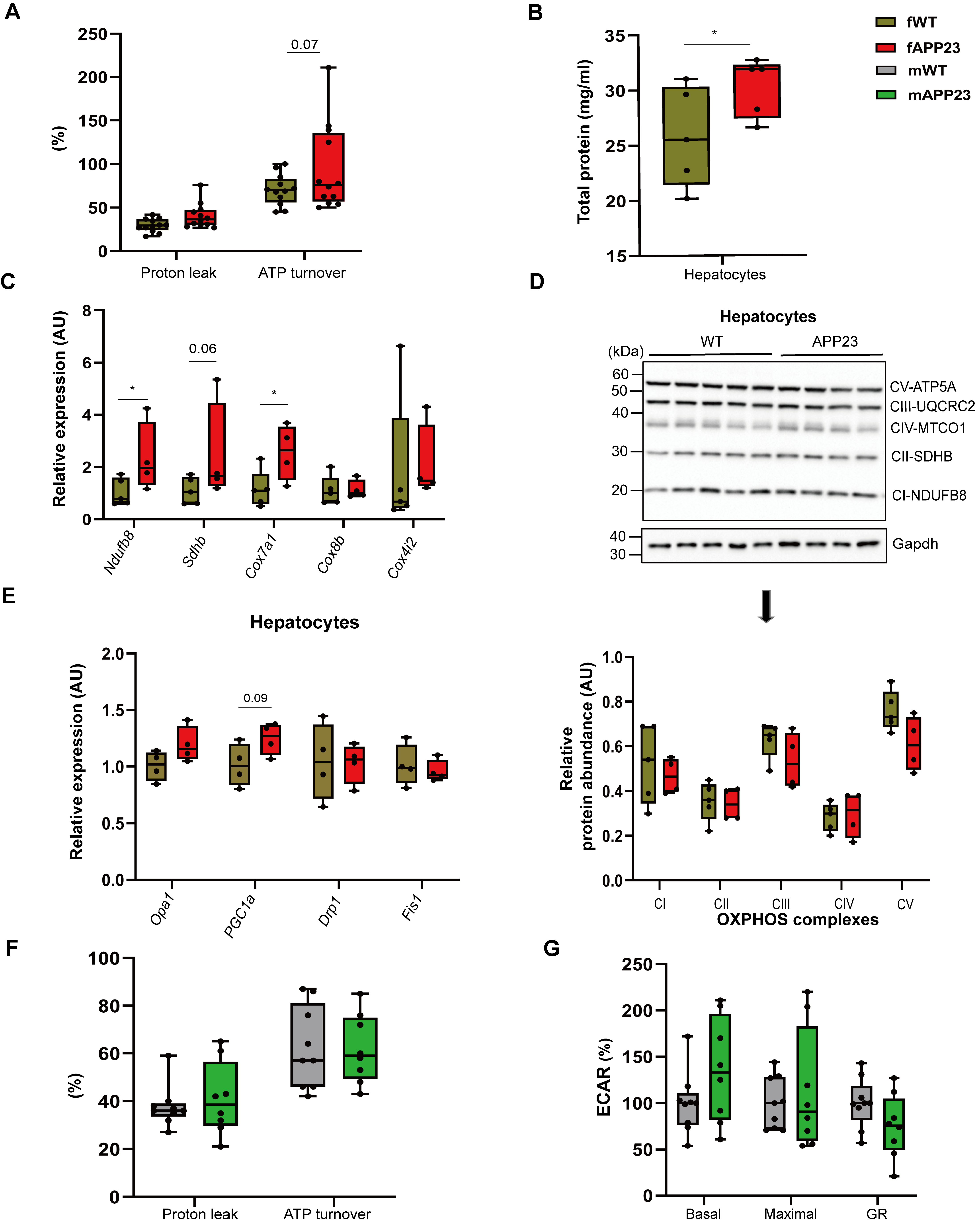
**

**Supplementary** Figure 4. **Protein, gene expression, OXPHOS and ECAR analysis of young APP23 mice. (A)** Proton leak and ATP turnover in hepatocytes from young females. (**B**) Total protein of 400.000 hepatocytes from young WT and APP23 females, n_female_ = 5/5. (**C**) Expression analysis of genes involved in mitochondrial oxidation in these hepatocytes, n_female_ = 5/4 for WT/APP23. (**D**) Representative western blots of key OXPHOS complexes and corresponding quantification in female hepatocytes normalized to GAPDH loading, n_female_ = 5/4. (**E**) Expression analysis of genes involved in mitochondrial biogenesis, mitochondria fusion and fuss quantified in hepatocytes from young mice, n_female_ = 5/4. (**F**-**G**) Proton leak and ATP turnover (**F**), ECAR (**G**) evaluation of primary hepatocytes from young males**.**, n_old_ = 5/4 for mixed-sex, n_male_ = 9/8 for WT/APP23. Data are presented as box plots (25^th^ to 75^th^ percentile) with median and whiskers from minimum to maximum and analyzed by unpaired one-tailed t-test with Welch correction (**B**), independent unpaired two-tailed t-test with Welch’s correction (**C**, **E** per group) or two-way ANOVA with Bonferroni multiple comparisons test (**A**, **D**, **F**-**G**). *p ≤ 0.05, AU: arbitrary units, FCCP: Trifluoromethoxy carbonylcyanide phenylhydrazone, GR: glycolytic reserve, OCR: oxygen consumption rate. R/A: rotenone + antimycin A.

**
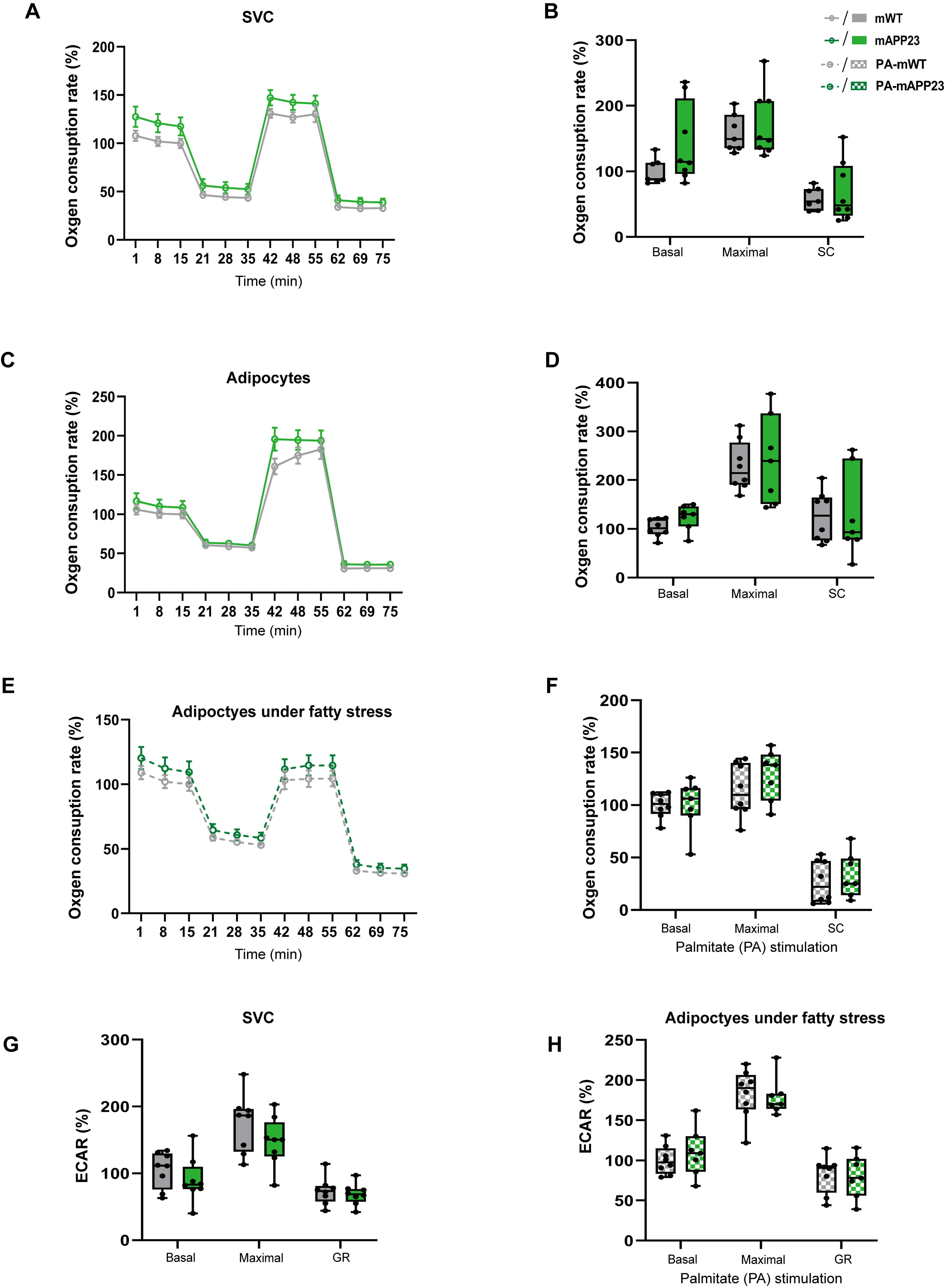
**

**Supplementary Figure 5. Mitochondrial respiration of SVC and differentiated adipocytes from young males during fatty acid stress**. (**A**-**B**) OCR and its calculation in SVC isolated from male eWAT, n_male_ = 8/8 for WT/APP23. (**C**-**F**) Comparative OCR analysis in adipocytes from young males under control (BSA-NaCl, **C**-**D**) or fatty acid stimulation (PA, 100 μM BSA-palmitate, **E**-**F**). (**G**-**H**) ECAR of unstimulated SVC (**G**) and adipocytes (**H**) under fatty acid stress. n_male_ = 8/7 for WT/APP23. 3 independent experiments, each with 5 technical replicates. Data are presented as mean ± SEM or box plots (25^th^ to 75^th^ percentile) with median and whiskers from minimum to maximum and analyzed by two-way ANOVA with Bonferroni multiple comparisons test (**B**, **D**, **F**-**H**). ECAR: extracellular acidification rate, eWAT: epididymal white adipose tissue, FCCP: Trifluoromethoxy carbonylcyanide phenylhydrazone, GR: glycolytic reserve, OCR: oxygen consumption rate, PA: BSA-palmitate, R/A: rotenone + antimycin A, SC: spare capacity, SVC: stromal vascular cells.


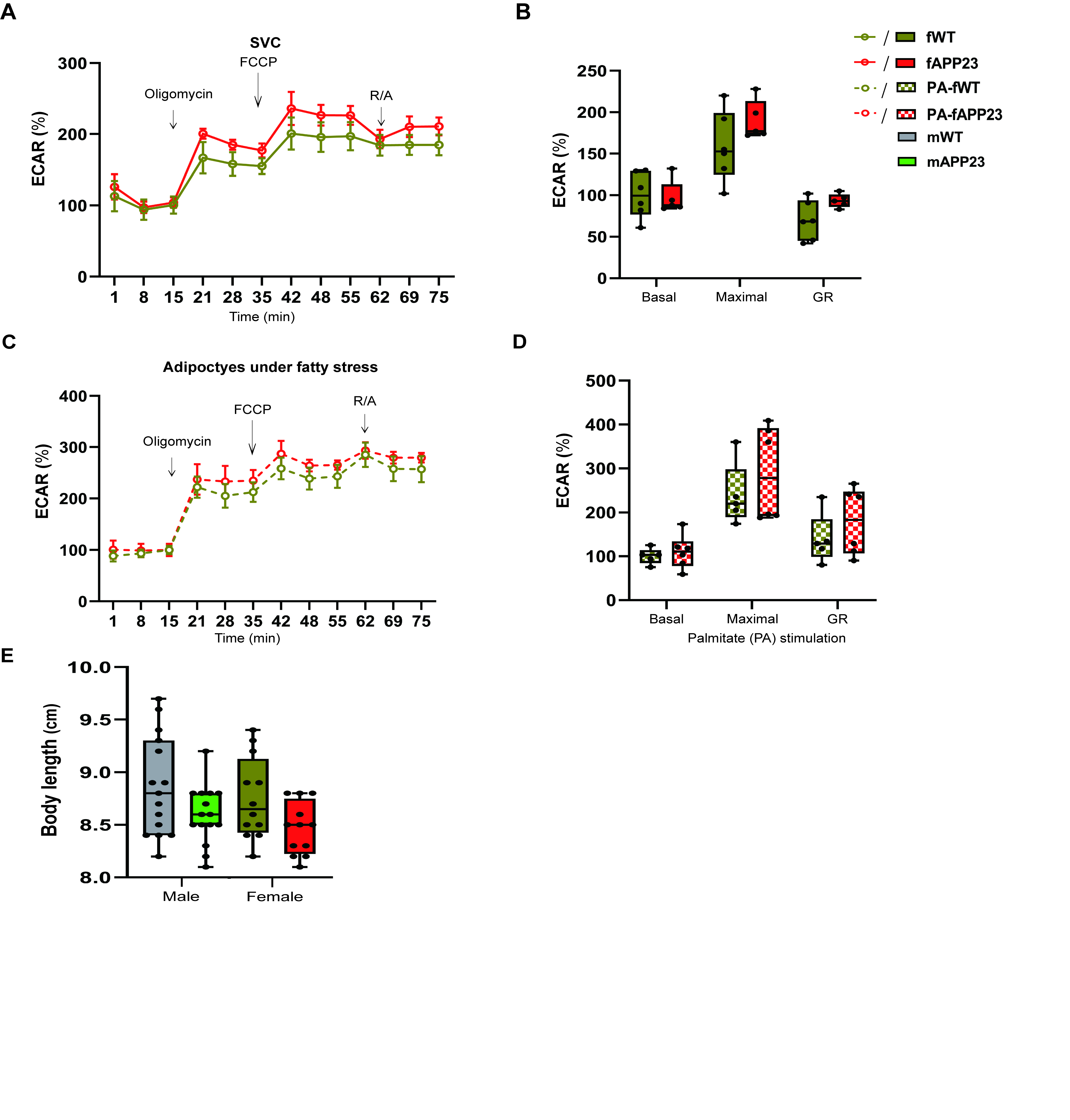


Supplementary Figure 6. ECAR of isolated SVC and differentiated adipocytes from young females under fatty acid stress. (**A**-**D**) ECAR curve and calculations of SVC (**A**-**B**) and *in vitro* differentiated adipocytes under fatty acid stimulation (PA, 100 μM BSA-palmitate, C-D). SVC: n_female_ = 6/5 for WT/APP23, PA: n_female_ = 6/6. 2 independent experiments, each with 5 technical replicates. (**E**) Body length of young male and female mice. n_male_ = 15/15, n_female_ = 12/12. Data are presented as mean ± SEM or box plots (25^th^ to 75^th^ percentile) with median and whiskers from minimum to maximum and analyzed by two-way ANOVA with Bonferroni multiple comparisons test (**B,** D, E). ECAR: extracellular acidification rate, FCCP: Trifluoromethoxy carbonylcyanide phenylhydrazone, GR: glycolytic reserve, PA: BSA-palmitate, R/A: rotenone + antimycin A, SVC: stromal vascular cells.

# Supplementary Tables

# Supplementary Table 1. Analyses of sex differences comparing males and female of the same genotype using three-way ANOVA with Bonferroni multiple comparisons test.

| **Referred data** | **Parameter** | **Genotype** | **p values** | | |
| --- | --- | --- | --- | --- | --- |
|  |  |  | **ANOVA** | **Bonferroni** | |
| Fig. 3 | Brain *APP23/App23* expression | WT | 0.25 | > 0.99 |  |
|  | Brain *APP23/App23* expression | APP23 |  | 0.24 |  |
| Fig. 3 | Liver *APP23/App23* expression | WT |  | > 0.99 |  |
|  | Liver *APP23/App23* expression | APP23 |  | > 0.99 |  |
| Fig. 3 | eWAT *APP23/App23* expression | WT |  | > 0.99 |  |
|  | eWAT *APP23/App23* expression | APP23 |  | > 0.99 |  |
| Fig. 3 | Brain *Mtor* expression | WT | 0.29 | > 0.99 |  |
|  | Brain *Mtor* expression | APP23 |  | > 0.99 |  |
| Fig. 3 | Brain *Cox2* expression | WT |  | 0.17 |  |
|  | Brain *Cox2* expression | APP23 |  | > 0.99 |  |
| Fig. 3 | Brain *Pgc1a* expression | WT |  | > 0.99 |  |
|  | Brain *Pgc1a* expression | APP23 |  | > 0.99 |  |
| Fig. 3 | Liver COX activity | WT | **< 0.001** | 0.06 |  |
|  | Liver COX activity | APP23 |  | **0.02** | * |
|  | Brain COX activity | WT |  | > 0.99 |  |
|  | Brain COX activity | APP23 |  | > 0.99 |  |
| Fig. 3 | Liver DCFDA ROS | WT | **< 0.001** | > 0.99 |  |
|  | Liver DCFDA ROS | APP23 |  | > 0.99 |  |
| Fig. 3 | Brain DCFDA ROS | WT |  | 0.10 |  |
|  | Brain DCFDA ROS | APP23 |  | 0.28 |  |
| Fig. 4 | Hepatocytes Basal OCR | WT | **< 0.001** | > 0.99 |  |
|  | Hepatocytes Basal OCR | APP23 |  | > 0.99 |  |
| Fig. 4 | Hepatocytes Maximal OCR | WT |  | 0.17 |  |
|  | Hepatocytes Maximal OCR | APP23 |  | **0.02** | * |
| Fig. 4 | Hepatocytes Spare capacity | WT |  | > 0.99 |  |
|  | Hepatocytes Spare capacity | APP23 |  | > 0.99 |  |
| Fig. 4 | Hepatocytes *Grp78* expression | WT | < **0.001** | > 0.99 |  |
|  | Hepatocytes *Grp78* expression | APP23 |  | > 0.99 |  |
| Fig. 4 | Hepatocytes *Atf6* expression | WT |  | > 0.99 |  |
|  | Hepatocytes *Atf6* expression | APP23 |  | > 0.99 |  |
| Fig. 4 | Hepatocytes *Sod1* expression | WT |  | > 0.99 |  |
|  | Hepatocytes *Sod1* expression | APP23 |  | > 0.99 |  |
| Fig. 5 | Adipocytes Basal ECAR | WT | **< 0.001** | > 0.99 |  |
|  | Adipocytes Basal ECAR | APP23 |  | > 0.99 |  |
| Fig. 5 | Adipocytes Maximal ECAR | WT |  | > 0.99 |  |
|  | Adipocytes Maximal ECAR | APP23 |  | > 0.99 |  |
| Fig. 5 | Adipocytes Glycolytic reserve | WT |  | > 0.99 |  |
|  | Adipocytes Glycolytic reserve | APP23 |  | > 0.99 |  |
| Fig. 6 | Adipocytes Basal OCR | WT | **< 0.001** | > 0.99 |  |
|  | Adipocytes Basal OCR | APP23 |  | > 0.99 |  |
| Fig. 6 | Adipocytes Maximal OCR | WT |  | 0.60 |  |
|  | Adipocytes Maximal OCR | APP23 |  | > 0.99 |  |
| Fig. 6 | Adipocytes Spare capacity | WT |  | 0.88 |  |
|  | Adipocytes Spare capacity | WT |  | 0.31 |  |
| Fig. 6 | SVC Basal OCR | WT | **< 0.001** | > 0.99 |  |
|  | SVC Basal OCR | APP23 |  | > 0.99 |  |
| Fig. 6 | SVC maximal OCR | WT |  | 0.47 |  |
|  | SVC maximal OCR | APP23 |  | **< 0.001** | *** |
| Fig. 6 | SVC Spare capacity | WT |  | 0.91 |  |
|  | SVC Spare capacity | APP23 |  | **< 0.001** | *** |
| Fig. 7 | Body weight | WT | **< 0.001** | **< 0.001** | *** |
|  | Body weight | APP23 |  | **< 0.001** | *** |
| Fig. 7 | Fat mass | WT |  | > 0.99 |  |
|  | Fat mass | APP23 |  | > 0.99 |  |
| Fig. 7 | Lean mass | WT |  | **< 0.001** | *** |
|  | Lean mass | APP23 |  | **< 0.001** | *** |
| Fig. 7 | Intake water | WT | **0.001** | 0.14 |  |
|  | Intake water | APP23 |  | > 0.99 |  |
| Fig. 7 | Intake diet | WT |  | > 0.99 |  |
|  | Intake diet | APP23 |  | 0.34 |  |

ECAR: extracellular acidification rate; eWAT: epigonadal adipose tissue; OCR: oxygen consumption rate; ROS: reactive oxidation species; SVC: stromal vascular cells. *p ≤ 0.05, ***p ≤ 0.001.

# Supplementary Table 2. Normal diet (ND, Rat/Mouse-Maintenance V1124-300, Ssniff).

| **Composition** | **%** | **Ingredients** |  |
| --- | --- | --- | --- |
| Carbohydrate | 67 kJ | **Minerals (%)** | **%** |
| Protein | 24 kJ | Calcium | 1.00 |
| Fat | 9 kJ | Phosphorus | 0.70 |
|  |  | Ca / P | 1.43 / 1 |
| **Crude Nutrients** | **%** | Sodium | 0.24 |
| Crude protein | 19.0 | Magnesium | 0.22 |
| **Ingredientes** |  | Potassium | 0.92 |
| **Amino acid** |  | **Fatty acids** |  |
| Lysine | 1.10 | C 12:0 | ---- |
| Methionine | 0.38 | C 14:0 | 0.01 |
| Cystine | 0.35 | C 16:0 | 0.45 |
| Met+Cys | 0.73 | C 18:0 | 0.09 |
| Threonine | 0.72 | C 20:0 | 0.01 |
| Tryptophan | 0.25 | C 16:1 | 0.01 |
| Arginine | 1.19 | C 18:1 | 0.62 |
| Histidine | 0.49 | C 18:2 | 1.76 |
| Valine | 0.92 | C 18:3 | 0.23 |
| Isoleucine | 0.79 | **Vitamins** | **Per kg** |
| Leucine | 1.39 | Vitamin A | 25000 IU |
| Phenylalanine | 0.89 | Vitamin D_3_ | 1500 IU |
| Phe+Tyr | 1.50 | Vitamin E | 135 mg |
| Glycine | 0.89 | Vitamin K (as MNB) | 20 mg |
| Glutamic acid | 4.22 | Thiamine (B1) | 86 mg |
| Aspartic acid | 1.84 | Riboflavin (B2) | 32 mg |
| Proline | 1.31 | Pyridoxine (B6) | 31 mg |
| Serine | 1.01 | Cobalamin (B12) | 150 ug |
| **Trace elements (mg)** | **Per kg** | Nicotinic acid | 153mg |
| Iron | 186 | Pantothenic acid | 59 mg |
| Manganese | 68 | Folic acid | 10 mg |
| Zinc | 91 | Biotin | 710 ug |
| Copper | 15 | Choline | 1370 mg |
| Iodine | 2.1 |  |  |
| Selenium | 0.3 |  |  |

# Supplementary Table 3. Summary of sample type, sex and genotype and sample size for each experiment.

| **Experiment** | **Sample** | **Sex & Genotype** | **n** |
| --- | --- | --- | --- |
| OCR & ECAR | Hepatocytes | fWT | 12 |
| OCR & ECAR | Hepatocytes | fAPP23 | 12 |
| OCR & ECAR | Hepatocytes | mWT | 9 |
| OCR & ECAR | Hepatocytes | mAPP23 | 8 |
| OCR & ECAR | Hepatocytes | oWT | 5 |
| OCR & ECAR | Hepatocytes | oAPP23 | 4 |
| OCR & ECAR | SVC | fWT | 6 |
| OCR & ECAR | SVC | fAPP23 | 5 |
| OCR & ECAR | SVC | mWT | 6 |
| OCR & ECAR | SVC | mAPP23 | 6 |
| OCR & ECAR | Adipocytes | fWT | 6 |
| OCR & ECAR | Adipocytes | fAPP23 | 6 |
| OCR & ECAR | Adipocytes | mWT | 9 |
| OCR & ECAR | Adipocytes | mAPP23 | 9 |
| OXPHOS Blots | Hepatocytes | fWT | 5 |
| OXPHOS Blots | Hepatocytes | fAPP23 | 4 |
| OXPHOS Blots | Hepatocytes | oWT | 5 |
| OXPHOS Blots | Hepatocytes | oAPP23 | 4 |
| qPCR | Hepatocytes | fWT | 5 |
| qPCR | Hepatocytes | fAPP23 | 4 |
| qPCR | Hepatocytes | oWT | 5 |
| qPCR | Hepatocytes | oAPP23 | 4 |
| TG | Plasma | fWT | 6 |
| TG | Plasma | fAPP23 | 6 |
| TG | Plasma | mWT | 5 |
| TG | Plasma | mAPP23 | 5 |
| TG | Liver | fWT | 6 |
| TG | Liver | fAPP23 | 6 |
| TG | Liver | mWT | 5 |
| TG | Liver | mAPP23 | 5 |
| BHB | Plasma | fWT | 7 |
| BHB | Plasma | fAPP23 | 9 |
| BHB | Plasma | mWT | 6 |
| BHB | Plasma | mAPP23 | 5 |
| BHB | Liver | fWT | 8 |
| BHB | Liver | fAPP23 | 7 |
| BHB | Liver | mWT | 10 |
| BHB | Liver | mAPP23 | 10 |
| NEFAs | Plasma | fWT | 9 |
| NEFAs | Plasma | fAPP23 | 9 |
| NEFAs | Plasma | mWT | 11 |
| NEFAs | Plasma | mAPP23 | 11 |
| Lipolysis | Adipose tissue | fWT | 6 |
| Lipolysis | Adipose tissue | fAPP23 | 6 |
| Lipolysis | Adipose tissue | mWT | 5 |
| Lipolysis | Adipose tissue | mAPP23 | 5 |
| Body weight | Mice | fWT | 9 |
| Body weight | Mice | fAPP23 | 9 |
| Body weight | Mice | mWT | 14 |
| Body weight | Mice | mAPP23 | 14 |
| Intake | Mice | fWT | 19 |
| Intake | Mice | fAPP23 | 15 |
| Intake | Mice | mWT | 17 |
| Intake | Mice | mAPP23 | 14 |
| Activity | Mice | fWT | 15 |
| Activity | Mice | fAPP23 | 9 |
| Activity | Mice | mWT | 8 |
| Activity | Mice | mAPP23 | 8 |
| Bomb calorimetry | ND Feces | mWT | 8 |
| Bomb calorimetry | ND Feces | mAPP23 | 7 |
| Bomb calorimetry | NCD Feces | fWT | 15 |
| Bomb calorimetry | NCD Feces | fAPP23 | 10 |
| Bomb calorimetry | HSD Feces | fWT | 10 |
| Bomb calorimetry | HSD Feces | fAPP23 | 14 |
| Bomb calorimetry | HFD Feces | fWT | 10 |
| Bomb calorimetry | HFD Feces | fAPP23 | 10 |
| qPCR | Liver | mWT | 7 |
| qPCR | Liver | mAPP23 | 4 |
| qPCR | Liver | fWT | 7 |
| qPCR | Liver | fAPP23 | 4 |
| qPCR | Brain | mWT | 7 |
| qPCR | Brain | mAPP23 | 4 |
| qPCR | Brain | fWT | 7 |
| qPCR | Brain | fAPP23 | 4 |
| Western blots | Liver | mWT | 7 |
| Western blots | Liver | mAPP23 | 4 |
| Western blots | Liver | fWT | 6 |
| Western blots | Liver | fAPP23 | 5 |
| Western blots | Brain | mWT | 7 |
| Western blots | Brain | mAPP23 | 4 |
| Western blots | Brain | fWT | 6 |
| Western blots | Brain | fAPP23 | 5 |
| DCFDA | Liver | mWT | 7 |
| DCFDA | Liver | mAPP23 | 4 |
| DCFDA | Liver | fWT | 6 |
| DCFDA | Liver | fAPP23 | 5 |
| COX activity | Liver | mWT | 7 |
| COX activity | Liver | mAPP23 | 4 |
| COX activity | Liver | fWT | 6 |
| COX activity | Liver | fAPP23 | 5 |
| DCFDA | Brain | mWT | 7 |
| DCFDA | Brain | mAPP23 | 4 |
| DCFDA | Brain | fWT | 6 |
| DCFDA | Brain | fAPP23 | 5 |
| COX activity | Brain | mWT | 7 |
| COX activity | Brain | mAPP23 | 4 |
| COX activity | Brain | fWT | 6 |
| COX activity | Brain | fAPP23 | 5 |

BHB: beta-hydroxybutyrate; DCFDA: 2´,7`-dichlorofluorescein diacetate; ECAR: extracellular acidification rate; fAPP23: female APP23 transgenic mice; fWT: female wild-type (WT) mice; HFD: high-fat diet; HSD: high-sucrose diet; mAPP23: male APP23 transgenic mice; mWT: male WT mice; NCD: normal-control diet; NEFA: non-esterified fatty acids; ND: normal diet; OCR: oxygen consumption rate; OXPHOS: oxidative phosphorylation; qPCR: quantitative real-time PCR; SVC: stromal vascular cells; TG: triglyceride.

# Supplementary Table 4. Cell culture media, materials and kits.

| **Materials and reagents** | **Company** |
| --- | --- |
| Earle’s Balanced Salt Solution | Gibco (Thermo) |
| Hanks’ Balanced Salt Solution | Gibco (Thermo) |
| Type IV collagenase/ Type I collagenase | Worthington Biochemical |
| Trypan blue | Sigma |
| Dulbecco’s Modified Eagle Medium | Gibco (Thermo) |
| Fetal bovine serum | Gibco (Thermo) |
| Penicillin-streptomycin | Gibco (Thermo) |
| Sodium pyruvate solution | Sigma |
| 3-Isobutyl-1-methylxanthine (IBMX) | AdipoGen |
| Dexamethasone | Sigma |
| TrypLE®-Express | Gibco (Thermo) |
| RevertAid-First-Stand-cDNA-Synthesis-Kit | Thermo |
| 1-Step-RT-qPCR system Master Mix | Promega-GoTaq® |
| BSA-palmitate | Cayman Chemical Company |
| Triglycerides FS 10’ kit | DiaSys |
| Hoechst 33342 staining kit | Thermo |
| DCFDA | Sigma |
| Total COX activity kit | Abcam |
| Methoxy-X04 | Merck |
| Trifluoromethoxy carbonylcyanide phenylhydrazone | Sigma |
| Oligomycin | Sigma |
| Rotenone + antimycin A | Sigma |

# Supplementary Table 5. Primers sequence for RT-PCR.

| **Gene** | **Orientation** | **Sequence** |
| --- | --- | --- |
| 18S ribosomal RNA (*18S*) | Fwd  Rev | 5-TTGACGGAAGGGCACCACCAG-3  5-GCACCACCACCCACGGAATCG-3 |
| Ribosomal protein L19 (*Rpl19*) | Fwd  Rev | 5-GGTGACCTGGATGAGAAGGA-3  5-TTCAGCTTGTGGATGTGCTC-3 |
| Peptidylprolyl isomerase A (*Ppia*) | Fwd  Rev | 5-TCAACCCCACCGTGTTCTTC-3  5-CCAGTGCTCAGAGCTCGAAA-3 |
| NADH dehydrogenase [ubiquinone] 1 beta subcomplex subunit 8 (*Ndufb8*) | Fwd  Rev | 5-GTTGCCGGGGTCATATCCTA-3  5-ACGGATCCCTCTCATGCTGT-3 |
| Succinate dehydrogenase [ubiquinone] iron-sulfur subunit (*Sdhb)* | Fwd  Rev | 5-TTTACCGATGGGACCCAGAC-3  5-CGTGTGCACGCCAGAGTATT-3 |
| Cytochrome c oxidase, subunit VIIa 1 (*Cox7a1)* | Fwd  Rev | 5-GCTCTGGTCCGGTCTTTTAGC-3  5-GTACTGGGAGGTCATTGTCGG-3 |
| Cytochrome c oxidase, subunit VIIIb (*Cox8b*) | Fwd  Rev | 5-TGTGGGGATCTCAGCCATAGT-3  5-AGTGGGCTAAGACCCATCCTG-3 |
| Succinate-Coenzyme A ligase, ADP-forming (*Cox4i2*) | Fwd  Rev | 5-CTGCCCGGAGTCTGGTAATG-3  5-CAGTCAACGTAGGGGGTCATC-3 |
| Transcript variant 1/Glucose related 78 (*Grp78*) | Fwd  Rev | 5-ACTTGGGGACCACCTATTCCT-3  5-ATCGCCAATCAGACGCTCC-3 |
| Activating transcription factor 6 1 (*Atf6*) | Fwd  Rev | 5-GACTCACCCATCCGAGTTGTG-3  5-CTCCCAGTCTTCATCTGGTCC-3 |
| Endoplasmic reticulum (ER) to nucleus signaling 1 (*Ire1*) | Fwd  Rev | 5-CACACCGACCACCGTATCTCA-3  5-CTCAGGATAATGGTAGCCATGTC-3 |
| Superoxide dismutase 1 (*Sod1*) | Fwd  Rev | 5-AACCAGTTGTGTTGTCAGGAC-3  5-CCACCATGTTTCTTAGAGTGAGG-3 |
| Erythroid derived 2 (*Nrf2*) | Fwd  Rev | 5-TAGATGACCATGAGTCGCTTGC-3  5-GCCAAACTTGCTCCATGTCC-3 |
| Kelch-like ECH-associated protein 1 (*Keap1*) | Fwd  Rev | 5-TGCCCCTGTGGTCAAAGTG-3  5-GGTTCGGTTACCGTCCTGC-3 |
| Cytochrome c oxidase subunit 1 (*Cox1*) | Fwd  Rev | 5-TGCCCCTGTGGTCAAAGTG-3  5-GGTTCGGTTACCGTCCTGC-3 |
| Cyclooxygenase-2 (*Cox2)* | Fwd  Rev | 5-TGAGCAACTATTCCAAACCAGC-3  5-GCACGTAGTCTTCGATCACTATC-3 |
| Dynamin-like GTPase OPA1 (*Opa1*) | Fwd  Rev | 5-TGGAAAATGGTTCGAGAGTCAG-3  5-CATTCCGTCTCTAGGTTAAAGCG-3 |
| Peroxisome proliferative activated receptor, gamma, coactivator 1 alpha *(Pgc1a)* | Fwd  Rev | 5-CCCAGGCAGTAGATCCTCTTCAA-3  5-CCTTTCGTGCTCATAGGCTTCATA-3 |
| Mechanistic target of rapamycin kinase *(Mtor)* | Fwd  Rev | 5-ACCGGCACACATTTGAAGAAG-3  5-CTCGTTGAGGATCAGCAAGG-3 |
| Dynamin-related protein 1 (*Drp1)* | Fwd  Rev | 5-ATGCCAGCAAGTCCACAGAA-3  5-TGTTCTCGGGCAGACAGTTT-3 |
| Mitochondrial fission 1 protein (*Fis1*) | Fwd  Rev | 5-CAAAGAGGAACAGCGGGACT-3  5-ACAGCCCTCGCACATACTTT-3 |
| (Human) amyloid precursor protein *(APP)* or murine *App* ***(G🡪A)*** | Fwd  Rev | 5-AGAAGGACAGACAGCACACC-3  5-TCATAACCTGGGACCGGATCT-3 |

Fwd: forward; Rev: reverse.
